# Implications of the Circumpolar Genetic Structure of Polar Bears for Their Conservation in a Rapidly Warming Arctic

**DOI:** 10.1371/journal.pone.0112021

**Published:** 2015-01-06

**Authors:** Elizabeth Peacock, Sarah A. Sonsthagen, Martyn E. Obbard, Andrei Boltunov, Eric V. Regehr, Nikita Ovsyanikov, Jon Aars, Stephen N. Atkinson, George K. Sage, Andrew G. Hope, Eve Zeyl, Lutz Bachmann, Dorothee Ehrich, Kim T. Scribner, Steven C. Amstrup, Stanislav Belikov, Erik W. Born, Andrew E. Derocher, Ian Stirling, Mitchell K. Taylor, Øystein Wiig, David Paetkau, Sandra L. Talbot

**Affiliations:** 1 Alaska Science Center, US Geological Survey, Anchorage, Alaska, United States of America; 2 Department of Environment, Government of Nunavut, Igloolik, Nunavut, Canada; 3 Ontario Ministry of Natural Resources and Forestry, Peterborough, Ontario, Canada; 4 All-Russian Research Institute for Nature Protection, Moscow, Russian Federation; 5 US Fish and Wildlife Service, Marine Mammals Management, Anchorage, Alaska, United States of America; 6 Wrangel Island State Nature Reserve, Moscow, Russian Federation; 7 Norwegian Polar Institute, Tromsø, Norway; 8 Natural History Museum, University of Oslo, Oslo, Norway; 9 Department of Zoology, Michigan State University, East Lansing, Michigan, United States of America; 10 Polar Bears International, Bozeman, Montana, United States of America; 11 Greenland Institute of Natural Resources, Copenhagen, Denmark; 12 Department of Biological Sciences, University of Alberta, Edmonton, Alberta, Canada; 13 Science & Technology Branch, Environment Canada, Edmonton, Alberta, Canada; 14 Faculty of Science and Environmental Studies, Lakehead University, Thunder Bay, Ontario, Canada; 15 Wildlife Genetics International, Nelson, British Columbia, Canada; Bangor University, United Kingdom

## Abstract

We provide an expansive analysis of polar bear (*Ursus maritimus*) circumpolar genetic variation during the last two decades of decline in their sea-ice habitat. We sought to evaluate whether their genetic diversity and structure have changed over this period of habitat decline, how their current genetic patterns compare with past patterns, and how genetic demography changed with ancient fluctuations in climate. Characterizing their circumpolar genetic structure using microsatellite data, we defined four clusters that largely correspond to current ecological and oceanographic factors: Eastern Polar Basin, Western Polar Basin, Canadian Archipelago and Southern Canada. We document evidence for recent (ca. last 1–3 generations) directional gene flow from Southern Canada and the Eastern Polar Basin towards the Canadian Archipelago, an area hypothesized to be a future refugium for polar bears as climate-induced habitat decline continues. Our data provide empirical evidence in support of this hypothesis. The direction of current gene flow differs from earlier patterns of gene flow in the Holocene. From analyses of mitochondrial DNA, the Canadian Archipelago cluster and the Barents Sea subpopulation within the Eastern Polar Basin cluster did not show signals of population expansion, suggesting these areas may have served also as past interglacial refugia. Mismatch analyses of mitochondrial DNA data from polar and the paraphyletic brown bear (*U. arctos*) uncovered offset signals in timing of population expansion between the two species, that are attributed to differential demographic responses to past climate cycling. Mitogenomic structure of polar bears was shallow and developed recently, in contrast to the multiple clades of brown bears. We found no genetic signatures of recent hybridization between the species in our large, circumpolar sample, suggesting that recently observed hybrids represent localized events. Documenting changes in subpopulation connectivity will allow polar nations to proactively adjust conservation actions to continuing decline in sea-ice habitat.

## Introduction

The distribution and viability of animal populations are dependent on the quantity, quality and connectivity of habitat. As habitat undergoes change, populations expand or become increasingly isolated. Because habitat change due to climate warming has been and is predicted to be most dramatic at the poles and especially for sea-ice habitat [1], Arctic species are expected to undergo dramatic shifts in distribution that may affect their viability [2]. Since 1979, the spatial extent of Arctic sea-ice in autumn has declined by over 9% per decade through 2010 [3]. Recent modeling predicts that nearly ice-free summers will characterize the Arctic before mid-century. However, as sea-ice loss is occurring “faster than forecasted” [3], the first nearly ice-free Arctic summer could occur as soon as 2016 [4]. The polar bear (*Ursus maritimus*), a specialist carnivore whose survival depends on sea-ice for foraging, migration and mating, is expected to be increasingly at risk due to changes in sea-ice habitat [5]. One modeling effort that included projections of future sea-ice conditions forecasted that two-thirds of the circumpolar population might be extirpated within half a century [6] unless greenhouse gas emissions are reduced and climate warming slowed [7]. These observations and expectations prompted the listing of the polar bear as a vulnerable species on the IUCN Red List in 2006 and as a threatened species in the United States in 2008. Subsequently, the 5 nations where polar bears occur are now developing a circumpolar management plan [8] outlining conservation actions and research needed to understand how the species will respond to changing habitat. Initial research has shown that decreasing metrics of sea-ice extent have been associated with declining polar bear body condition [9]–[11], survival [12]–[14] and population size [13], although effects of sea-ice decline on polar bears are variable [15], [16]. Using global circulation models (GCMs) and polar bear habitat-use data, Durner et al. [17] predicted that mean summer polar bear optimal habitat in the Polar Basin (approximately 50% of the most northerly portion of polar bear range) will decrease by 68% by the end of the 21st century. Although with “faster than forecasted” observations of the rate of sea-ice reduction [3], [4], this timeline will likely be compressed. At the scale of the polar bear's entire circumpolar range, changes in sea-ice phenology and quality are predicted to influence the demographics [6], [18], connectivity [19], [20], degree of genetic isolation [21], and therefore viability, of the 19 global semi-discrete subpopulations recognized by the IUCN/Polar Bear Specialist Group [22].

Though movement of individual polar bears across present-day subpopulation boundaries is evident from capture-recapture data and hunter tag returns [23], satellite telemetry [24]–[26] and genetic surveys [21], [27], [28], the IUCN subpopulation boundaries were originally developed based on patterns of sea-ice formation and melt, and observations of polar bear seasonal fidelity. Though polar bears are currently thought to constitute a panmictic, single evolutionary unit [27], reduced range and habitat connectivity may increasingly fragment subpopulations resulting in transient refugia and meta-population dynamics. Some subpopulations may become more productive, whereas others may become less viable [5]. There is already evidence of change in the contemporary distribution of polar bears. For example, polar bears, once common in Newfoundland [29], are now seen there only infrequently and in small numbers. Similarly, polar bears once regularly summered on St. Lawrence and St. Matthew islands in the Bering Sea [30]–[32]. Now they are irregularly observed in the Bering Sea and do not spend summers on St. Matthew Island. Although these changes in polar bear distribution may also have been related to overharvest, the recent reductions in the extent of sea-ice due would prevent current and regular use of these areas.

Future responses of polar bears to declining habitat may mirror their responses during past fluctuations in climate. Paleodemographic genetic estimates using mitochondrial DNA (mtDNA) have suggested that polar bear evolution and effective population size have tracked key climate events, similar to the paraphyletic brown bear (*U. arctos*). Both species experienced fluctuations in effective population size throughout their history, including prolonged declines over the past 500,000 years, although this decline is more pronounced in polar bears [33]. Population fluctuations of brown and polar bears appear to be offset, with population size in brown bears decreasing, and in polar bears increasing, during Pleistocene cooling events [33]. This offset is consistent with observations from other paired boreal and Arctic species, including voles (*Microtus* spp.) and shrews (*Sorex* spp.; [34]). Periodic hybridization between the two species of bears has resulted in conflicting estimates of divergence time, presence of polar bear-like mtDNA in certain extant [35]–[37] and extinct [38]–[40] brown bear populations, and long stretches of polar bear nuclear DNA in the brown bear genome [33], [41]. Signatures of admixture are consistent with overlapping distributional ranges (and habitat) of both species in some areas, and viable hybrids recently have been documented in wild populations [42]. The rate and periodicity of hybridization events through time may result from shifting ranges of one or both species in response to climatic change [33]. Hence, connectivity among subpopulations of polar bears, and interactions between polar bears and brown bears, will continue to change during the current period of sea-ice decline.

In this paper, we use mtDNA and neutral microsatellite DNA data to establish the historical and current genetic structure and gene flow dynamics in polar bears across their entire circumpolar range, and specifically among the subpopulation units that are used for polar bear management. We employ clustering techniques [43], [44] to evaluate underlying circumpolar structure, which may be cryptic and not necessarily related to subpopulation boundaries [45]. By using coalescent theory [46], we examine historical (mtDNA) and modern (microsatellite) asymmetries in gene flow among clusters of subpopulations to determine the underlying connectivity. We assess symmetry in gene flow during the period of very recent climate warming and ice habitat decline with Bayesian methods [47]. We also directly compare polar bear samples from the 1980s through the 2010s (over ∼2–3 generations), to test for temporal shifts in genetic structure within subpopulations. We further determine the extent of sex-bias in gene flow as a mechanism underlying connectivity of subpopulations of polar bears. We compare the polar bear mtDNA data to similar data in the brown bear, to provide context to the analyses of phylogenetic structure, historical population fluctuations and gene flow in polar bears during historical climate fluctuations. We also address recent hypotheses about the relationship between modern polar bears and brown bears [33], [40], [41]. Our aim is to provide a comprehensive baseline of historical and contemporary genetic structure for polar bears, throughout their circumpolar range, which will complement ecological and evolutionary research, provide a framework for effective conservation actions [48], [49], and provide a geographically comprehensive baseline for future assessment and management.

## Materials and Methods

### Ethics Statement

Data were used from samples collected in previously permitted research studies between 1973 and 2006 (n = 869; S11 and S12 Tables; [27], [28]). We also newly extracted DNA from polar bear tissue collected for ecological research studies permitted by various jurisdictions (S11 Table; details of permits in S12 Table). Shed polar bear hair samples from the Laptev Sea (LP) and Kara Sea (KS) subpopulations were collected from the ground by Russian biologists, and did not require permitting for collection. Other polar bear samples were collected from the legal harvest in the territory of Nunavut, Canada (S12 Table). No polar bears were harvested or captured for the purpose of this study.

### Sampling

We analyzed DNA from 2,748 polar bear samples from 18 subpopulations at 16–21 microsatellite loci (depending on subpopulation), and 411 samples from 15 subpopulations at the mtDNA control region (S1 Fig. a,b). Some samples were used for both mtDNA and microsatellite analyses. We collected tissue samples of polar bears year-round from sport or subsistence harvest (n = 198), or capture operations (n = 2410) in all countries that have polar bears: Canada, Greenland, Norway, Russia and the United States. Remotely-collected biopsies from Canada (n = 65) and shed hair from Russia (n = 36; S11 Table) were also used. Samples were collected from 18 of the 19 recognized subpopulations of polar bears [22] (S1a,b Fig.): Baffin Bay (BB); Barents Sea (BS); Chukchi Sea (CS); Davis Strait (DS); East Greenland (EG); Foxe Basin (FB); Gulf of Boothia (GB); Kane Basin (KB); KS; LP; Lancaster Sound (LS); M'Clintock Channel (MC); Northern Beaufort Sea (NB); Norwegian Bay (NW); Southern Beaufort Sea (SB); Southern Hudson Bay (SH); Viscount Melville (VM); and Western Hudson Bay (WH). The 19^th^ subpopulation – the Arctic Basin – is an inaccessible, unmanaged subpopulation, and is thought not to currently function as a year-round habitat. Only samples from independent polar bears (i.e., >2 years) were used, unless the accompanying mother and/or sibling were not already in the sample set. Ninety-seven percent of the samples had known latitude and longitude (S1a,b Fig.), but analyses were conducted on all samples. For additional details, see S1 Supporting Information: Details of materials and methods, Sampling.

### Laboratory Methods

DNA extraction, PCR amplification, and genotyping or sequencing protocols for microsatellite and mtDNA data of ursids are described in the literature [27], [28], [50]. For additional details, see S1 Supporting Information: Details of materials and methods, Laboratory Techniques.

### Genetic Diversity

We quantified genetic variation and tested for neutrality in both microsatellite and mtDNA data using a variety of computer programs routinely used to analyze genetic data [51]–[59]. For additional details, see S1 Supporting Information: Details of materials and methods, Genetic Diversity.

### Decadal Comparisons

We used microsatellite data to test for differences in the distribution of allele frequencies [54] between decadal groups for each of nine polar bear subpopulations for which we had data for multiple decades (S1a Fig.): SB (n = 83 (1980s), 45 (1990s), 64 (2000s) and 41 (2010s)); CS (n = 30 (1980s), 27 (1990s), 71 (2000s) and 138 (2010s)); FB (n = 30 (1990s), 89 (2000s)); BB (n = 48 (1990s) and 126 (2000s)); WH (n = 24 (1980s), 22 (2008)); GB (n = 30 (1990s), 15 (2008)); LS (n = 30 (1980s–90s), 31 (2000s); the Labrador portion of DS (n = 27 (1990s) and 217 (2000s)); and the Svalbard region of BS (n = 192 (1990s) and 249 (2000s)). We also used Bayesian clustering [60] to infer the occurrence of population structure among groups sampled during different decades. For additional details, see S1 Supporting Information: Details of materials and methods, Decadal Comparisons.

### Genetic Differentiation

We assessed genetic differentiation among the subpopulations of polar bears using both microsatellite and mtDNA data. Overall standardized estimates of F_ST_ variance based on microsatellite loci were calculated [55], [61], as were estimates of inter-subpopulation variance [55]; significance was based on random permutation tests. We also addressed previous hypotheses about within-subpopulation structuring within DS [15] and SH [21], using the same tests. For mtDNA data, we applied the evolutionary model that best fit mtDNA [62] to calculate Φ [63], and tested for significance [55]. We used hierarchical analyses of molecular variance (AMOVA; [55]) with both marker types to test for significance of geographic partitioning of hypothesized genetic units. For additional details, see S1 Supporting Information: Details of materials and methods, Genetic Differentiation.

### Sex-specific Philopatry

To assess sex-specific philopatry and gene flow of polar bears, we plotted pairwise F_ST_ values from microsatellite (biparental inheritance) and mtDNA (matrilineal inheritance) data of each subpopulation, calculated after accounting for differences in the effective size between the two genomes (*F*_ST_(nu) = 1–*e* ^0.25*ln[1–*F*ST(mt)]^) [64]. For interspecific comparison, we plotted these values with pairwise values generated from populations of brown bears from southeast Alaska [50].

### Modern Circumpolar Structure

We examined the modern structuring of the circumpolar polar bear population based on microsatellite data using Bayesian clustering methods [65] implemented in structure version 2.0 [43]. We considered advice from Evanno et al. [66] and Pritchard et al. [43] to determine the most likely number of genetic clusters of polar bears, in addition to biological rationale. For significance testing, all α-values were set at 0.05 and, where appropriate, adjusted using Bonferroni procedures [67].

### Gene Flow

We estimated modern (microsatellite) and historical (mtDNA) gene flow between clusters that we had previously identified with microsatellite DNA. Estimates of gene flow among the clusters of polar bears were calculated for microsatellite loci using bayesass, version 3.0.1 [43], [65]. The number of migrants per generation (*N_e_m*) for nuclear microsatellite and number of female migrants per generation (*N_f_m*) for mtDNA were calculated using migrate version 3.0.3 [46], [65], [68]. For additional details, see S1 Supporting Information: Details of materials and methods, Gene Flow.

### Phylogenetic Analyses of MtDNA Sequences

Using mtDNA data, we made phylogenetic comparisons among haplotypes found in 15 subpopulations of polar bears. To root the phylogenetic tree, we included 37 haplotypes from 144 individuals representing the three Alaskan brown bear clades [37]. Phylogenetic analyses of the polar and brown bear control region sequences were conducted using paup*4.0b8 [69], using maximum parsimony (MP), maximum likelihood (ML) and distance (minimum evolution, ME) approaches. For additional details, see S1 Supporting Information: Details of materials and methods, Phylogenetic Analyses of MtDNA Sequences.

### Changes in Historical Population Size

Using the mtDNA dataset, we assessed historical signatures of population growth/expansion within subpopulations and/or within larger regional groupings of polar bears using: extended Bayesian skyline plots [70]; mismatch distributions [71] and their raggedness (*rg*; [72]); the shape of phylogenetic trees; neutrality tests sensitive to population fluctuations [73], [74]; comparison of diversity indices (haplotype diversity [*h*] and nucleotide diversity [π]); and coalescent-based simulation methods [75]. Significance of expansion measures was tested via coalescent simulations [76]. To estimate the time since population expansion, we used mismatch distributions and the nonlinear least-squares approach. For estimation of mutation rate we used a coalescent Bayesian framework and included control region haplotype sequences of representative brown and polar bears [77]. Parameters were set in beauti, part of the v1.6.1 software package [78]. For additional details, see S1 Supporting Information: Details of materials and methods, Changes in Historical Population Size.

## Results

### Genetic Diversity

We collected multi-locus microsatellite genotypes from samples of 2,748 polar bears from 18 of 19 circumpolar polar bear subpopulations (data are deposited at datadryad.org, doi:10.5061/dryad.v2j1r). Both allelic richness (range, 5.06 to 5.94) and observed heterozygosity (H_o_; range, 0.64 to 0.73) were similar across subpopulations (S1 Table). Overall H_o_ was 0.70. Significant departures from Hardy-Weinberg Equilibrium (HWE) were detected in 5 of 338 cases (locus by subpopulation), with no individual locus or subpopulation accounting for a disproportionate number of significant test results. Accordingly, no loci were dropped from further analyses due to deviation from HWE. Overall, significant (*P*<0.05) associations of alleles between markers (linkage disequilibrium, LD) were identified for 47 of 210 pairs of loci (10.5 positive tests would be expected due to Type I error), suggesting that the global population has significant admixture or substructuring. LD patterns were absent with the exception of one pair of loci, suggesting that the observed LD was due to population structure rather than physical linkage on the chromosome. LD within the DS subpopulation, especially in association with, but not isolated to, locus G10X, produced 30 of 67 significant (*P*<0.002) test results. That the LD was relatively restricted to one subpopulation is suggestive of significant population substructuring within DS.

**Figure 1:**
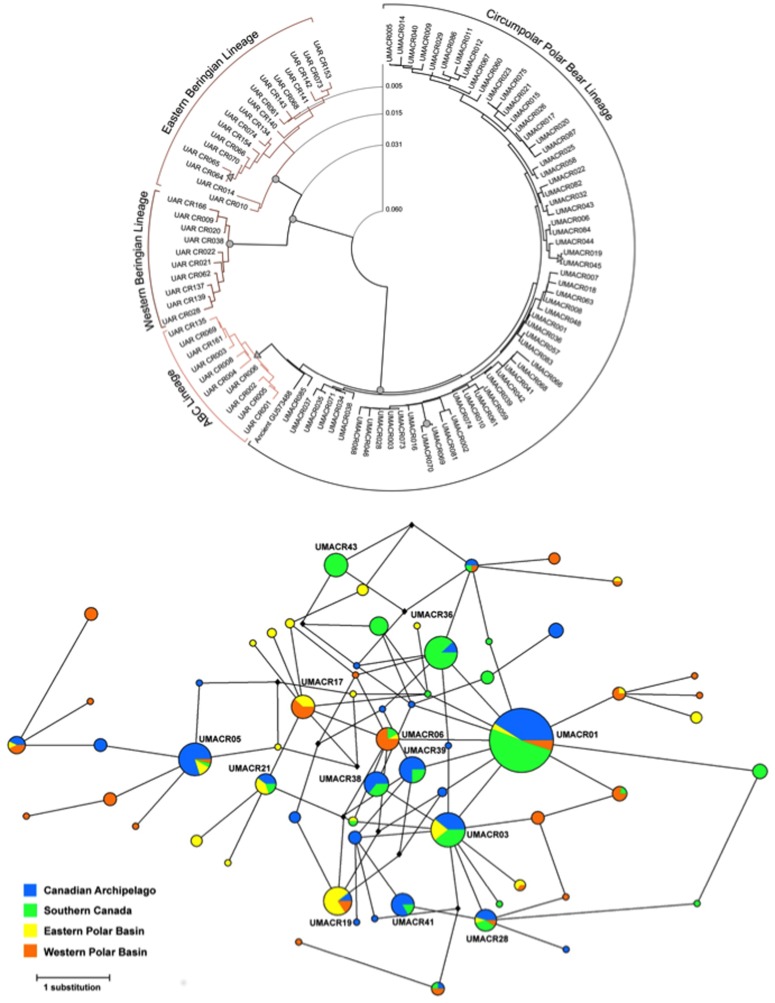
Relationships between mitochondrial haplotypes of polar bears from the circumpolar range (15 subpopulations). **a**. Minimum evolution tree showing the relationships between 63 mitochondrial DNA control region haplotypes for polar bears from these subpopulations, the ancient Poolepynten (GenBank Accession No. GU573488) polar bear and haplotypes found within the three clades of Alaskan brown bears (GenBank Accession No. KM821364–KM821401). Numbers represent distances between deeper nodes, under the Tamura-Nei distance (I+G_0.69_) model. Filled circles indicate nodes with>70% bootstrap support, and arrows at nodes indicate 50–69% bootstrap support. **b**. Unrooted 95% parsimony network showing relationships of the 64 haplotypes. The size of the node corresponds to the frequency of each haplotype (numbered) with black squares representing unsampled haplotypes.

Analysis of 411 polar bears from 15 subpopulations (Fig. 1a,b; S1 Table) identified 63 mtDNA control region haplotypes characterized by 35 polymorphic sites, including 31 transitions, one transversion and three insertion/deletions (S2 Table). Haplotype diversity (*h*) ranged from 0.00 to 0.95 and nucleotide diversity (π) ranged from 0.000 to 0.007 (S1 Table). Private haplotypes were observed in the four clusters identified with microsatellite DNA (below in *Genetic Differentiation of Subpopulations*), though most private haplotypes were only represented by a single or few individuals (Fig. 1b). Haplotype sequences have been submitted to GenBank (S2 Table). There was no signal of deviation from selective neutrality for any of the 15 subpopulations (Eν = 0.091–0.444, *P* = 0.303–0.990; S1 Table).

### Decadal Comparisons

We evaluated genetic differentiation at microsatellite markers within nine subpopulations between samples collected in early and late time periods (S3 Table). We found no significant levels of genic and genotypic differentiation and substructure between early and late time periods (S3 Table). Thus, all data were pooled within each subpopulation for subsequent analyses.

### Genetic Differentiation of Subpopulations: Microsatellite Data

The circumpolar estimate of population genetic structure across the 18 subpopulations was significant (F_ST_ = 0.034; 95% CI: 0.027–0.043). The upper limit of F_ST_ for this data set, taking into account the level of genetic diversity, is 0.295, therefore our overall estimate accounts for 11.5% of the maximum possible level of genetic structure. Thirty-one (20%) of 153 possible pairwise comparisons among subpopulations showed a signal of significant differentiation (adjusted for multiple-comparisons) based on at least one of four metrics (mean F_ST_, R_ST_, genic and/or genotypic differentiation; S4 and S5 Tables; genotypic differentiation was assessed for those subpopulations out of HWE). For example, WH exhibited genetic differentiation from most other subpopulations, with 11 of 17 possible comparisons showing significant differentiation with all metrics. In contrast, BS was genetically similar to most other subpopulations, and was typically grouped with EG, KS and LP. Significant genetic structure was observed within DS and SH, indicating further regional structuring within these subpopulations (S6 Table). Using the Fischer's Exact test and α = 0.05 for comparison to Paetkau et al. [27] who found 98% of the 120 pairwise comparisons significant, 150 of our 153 possible tests (98%) were significant. Using this latter test, the three pairs of subpopulations that did not show differentiation from each other were MC and LS, KS and LP, and KS and BS.

**Figure 2:**
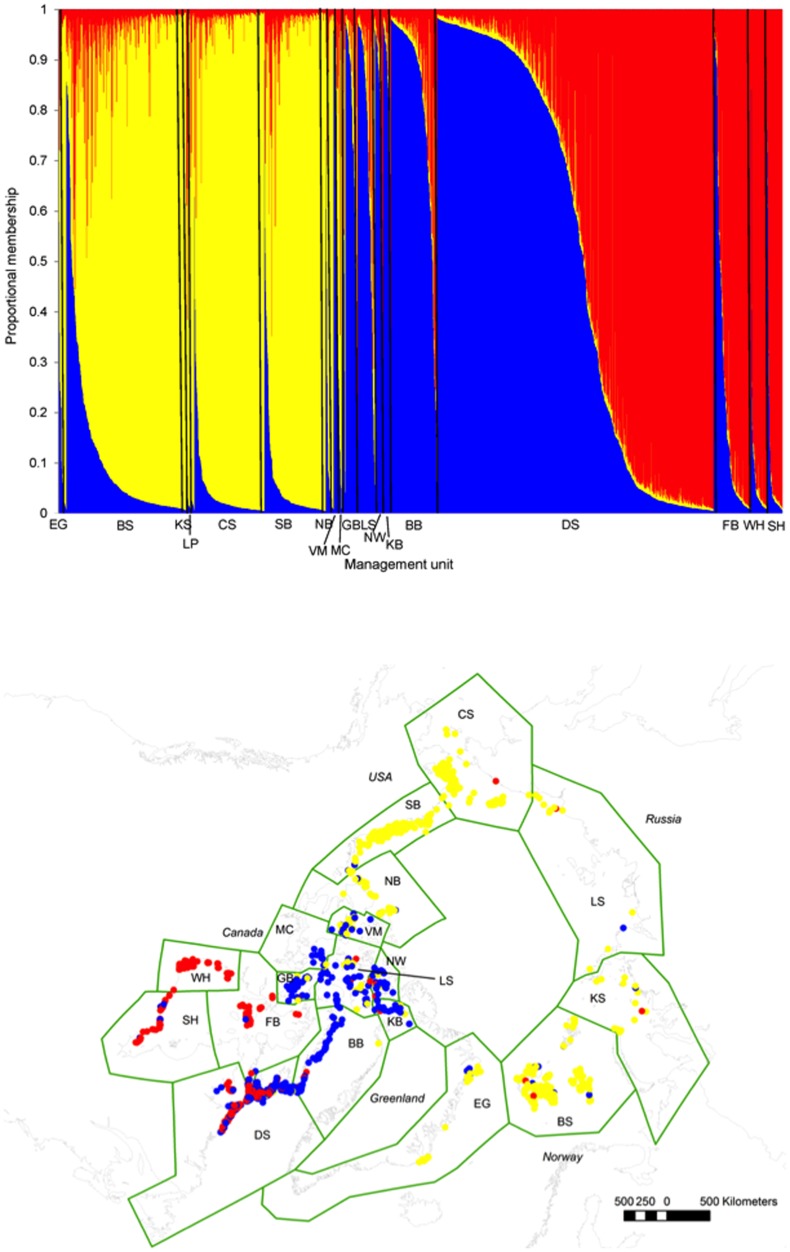
Assignment of individual polar bears (S11 Table) from their circumpolar range (19 subpopulations) to regional genetic clusters. **a**. structure [43] assignment plot for microsatellite signatures (n = 2,899) of polar bears. Y-axis represents proportional membership each of three most-likely groups identified by program structure (Southern Canada [red dots], Canadian Archipelago [blue dots] and the Polar Basin [yellow dots]). Note, based on subsequent analysis (S2c Fig., S6 Table) we discuss the Polar Basin cluster as two groups: the Eastern Polar Basin Western Polar Basin clusters. Individuals are organized (each represented by a single vertical line) along the X-axis according to subpopulation: East Greenland (EG), Barents Sea (BS); Kara Sea (KS); Laptev Sea (LP); Chukchi Sea (CS); Southern Beaufort Sea (SB); Northern Beaufort Sea (NB); Viscount Melville (VM); M'Clintock Channel (MC); Gulf of Boothia (GB); Lancaster Sound (LS); Norwegian Bay (NW); Kane Basin (KB); Baffin Bay (BB); Davis Strait (DS); Foxe Basin (FB); Western Hudson Bay (WH) and Southern Hudson Bay (SH). Individuals within each subpopulation are arranged according membership to one of the three clusters. **b**. Geographical locations of (n = 2,650) samples in the three genetic clusters.

Using the polar bear microsatellite data and the program structure [43], ΔK was maximized when K (i.e., number of likely clusters) = 2 (ΔK = 577.7; S2 Fig.). According to this model, polar bears residing in the Polar Basin were assigned to one cluster and all other polar bears were assigned to another. The second highest ΔK was with K = 3 (ΔK = 263.2), which partitioned polar bears residing in the Canadian Archipelago and Southern Canada into their own clusters, with the third cluster in the Polar Basin (Fig. 2a,b); K = 3 was identified as the most likely clustering pattern using Pritchard et al.'s [43] criteria. Regional sub-structuring also was uncovered within higher hierarchical clusters (in the K = 3 scenario), but only significant within the Polar Basin. Within the Polar Basin, polar bears were further separated into Eastern and Western Polar Basin clusters (K = 2, ΔK = 271.2; S3 Fig. c). The analyses within Canadian Archipelago Cluster (K = 2, ΔK = 255.7) and Southern Canada Cluster (K = 2, ΔK = 350.5) did not result in a discrete geographical pattern similar to that observed within the Polar Basin (S3a,b Figs.). The split found within the Polar Basin group reflects some known ice patterns (see Discussion) and thus we decided to group polar bears into four clusters for subsequent analyses: Eastern Polar Basin, Western Polar Basin, Canadian Archipelago and Southern Canada.

Using AMOVA, we rejected all but one hypothesis of among-group variance in microsatellite allele frequencies, which placed polar bear subpopulations into three clusters (Polar Basin, Canadian Archipelago, and Southern Canada; S7 Table).

### Genetic Differentiation of Subpopulations: MtDNA data

We observed significant global variance in mtDNA haplotypic diversity (Φ*_ST_* = 0.210; *P*<0.001). Population pairwise Φ*_ST_* values ranged from −0.046 to 0.805, and 77 of 105 pairwise comparisons were significant (S5 Table). We detected significant differences in the distribution of haplotypes (χ2 _df = 3_ = 13.21–∞, *P*<0.002, α = 0.05) in all comparisons that involved subpopulations characterized by three or fewer mtDNA samples (MC, VM and NW). In 33 of 39 comparisons involving these three subpopulations, we rejected the null hypothesis of equal distribution of haplotypes (S5 Table).

Significant mean Φ_CT_ values were observed for all AMOVA hypotheses based on mtDNA data (i.e., historical structure and sex specific dispersal). Among these, the hypothesis that grouped subpopulations into two broad geographic regions (the Polar Basin, and Canada's eastern Arctic and Subarctic) yielded the highest mean Φ_CT_ value (S7 Table).

Mitogenomic structure for polar bears was less than the partitioning observed among brown bear populations separated by similar geographic distances (S4 Fig.). Pairwise comparisons among subpopulations revealed that genetic structure at nuclear markers was typically less than expected given the genetic structure observed in mtDNA (S4 Fig.).

### Gene Flow

**Figure 3:**
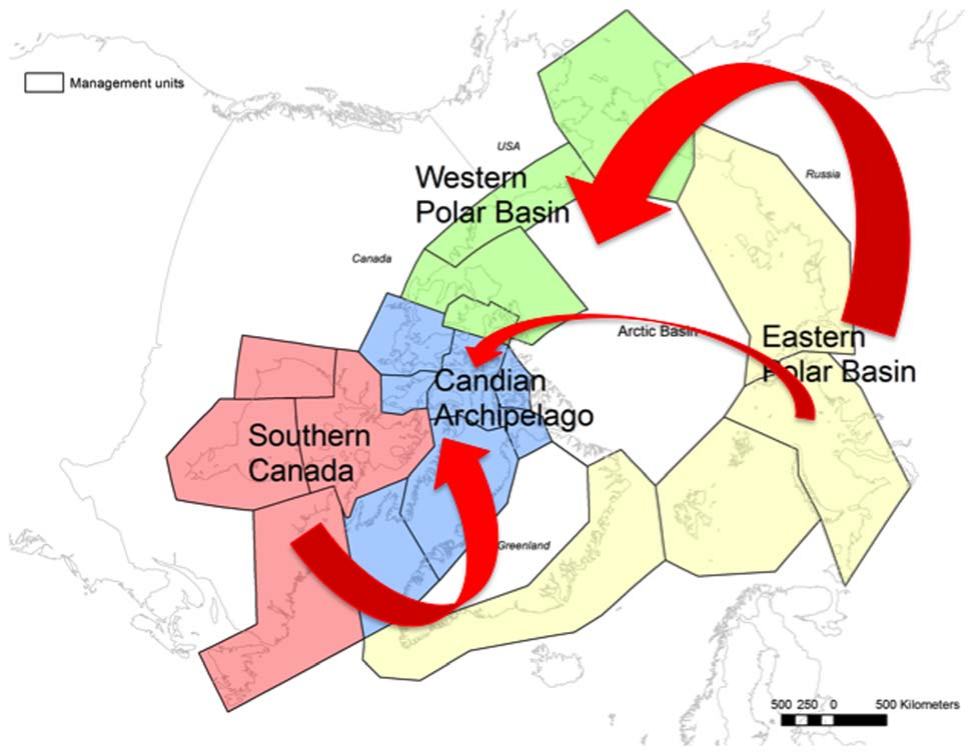
Recent directional gene flow (ca. 3–10 generations) calculated on the basis of allelic frequencies (number of migrants, *m*) among polar bear clusters. Data generated using the program bayesass [47], examining gene flow relationships between the four clusters of polar bears (Southern Canada (SC; red), Canadian Archipelago (CA; blue), Eastern Polar Basin (EP; yellow) and Western Polar Basin (WP; green)), identified by program structure analysis of microsatellite data. Arrow widths represent only directional gene flow values that are significantly different from zero (no migration) and from the value for migration in the opposite direction.

Asymmetrical gene flow was inferred among the four clusters (Easter Polar Basin, Western Polar Basin, Canadian Archipelago, Southern Canada) across both marker types and all analyses. In the microsatellite data set, biases in the directionality of gene flow estimated using the coalescent were not as strong as those estimated using allelic frequency (Table 1; Fig. 3). Microsatellite data analyzed using allelic frequencies (i.e., past 1–3 generations) indicated directional gene flow into the Canadian Archipelago cluster from the other clusters (except from the Western Polar Basin), as well as from the Eastern Polar Basin into the Western Polar Basin (Table 1; Fig. 3). The Eastern Polar Basin and Southern Canada clusters were represented by a high proportion of “non-migrant” individuals, suggesting these regions are sources of dispersal (S8 Table). Coalescent-based estimates of the microsatellite data revealed asymmetrical gene flow from Western Polar Basin cluster into the Canadian Archipelago cluster, with all other comparisons suggesting symmetrical gene flow as indicated by overlapping 95% confidence limits (Table 1).

**Table 1:** Directional gene flow estimated based on allelic frequency (proportion of non-migrants, *m*) in bayesass and based on the coalescent (effective number of migrants DNA *N_e_m* and female migrants *N_f_m* per generation) in migrate between four clusters of polar bears calculated from microsatellite and mitochondrial control region data.

Cluster pair	Nuclear DNA: 1–3 gen. Proportion of migrants (%)			Nuclear DNA: within Holocene *N_e_m*			Mitochondrial DNA: inclusive of Pleistocene *N_f_m*		
	Immigration	Emigration	Dir*	Immigration	Emigration	Dir	Immigration	Emigration	Dir
EP									
–WP	0.9 (0.0–2.5)	29.0 (24.9–33.2)	Source	3.3 (2.8–3.8)	3.1 (2.7–3.6)	–	3.5 (1.3–5.8)	1.2 (0.9–2.9)	–
–CA	2.7 (0.0–6.9)	15.5 (7.4–23.5)	Source	3.0 (2.6–3.5)	2.6 (2.3–3.0)	–	2.1 (1.4–7.7)	1.8 (1.0–2.9)	–
–SC	2.4 (0.0–5.9)	1.8 (0.0–4.4)	–	2.7 (2.2–3.2)	2.7 (2.3–3.1)	–	1.2 (0.8–4.2)	0.3 (0.2–0.8)	Sink
WP									
–CA	2.1 (0.0–5.3)	0.5 (0.0–1.6)	–	3.2 (2.7–3.7)	4.3 (3.8–4.9)	Source	2.9 (1.4–4.9)	0.9 (0.7–1.7)	–
–SC	1.1 (0.0–3.1)	0.5 (0.0–1.5)	–	2.5 (2.1–2.9)	2.1 (1.8–2.4)	–	1.2 (0.9–2.9)	0.2 (0.2–0.6)	Sink
CA									
–SC	14.1 (6.9–21.2)	2.6 (0.0–5.8)	Sink	2.5 (2.2–2.9)	2.5 (2.1–2.9)	–	1.5 (0.8–2.5)	0.5 (0.4–1.1)	–

^*^Gene-flow estimates are listed as immigration into population A from population B and emigration from population A into population B. For example, gene flow between WP and CA with microsatellite loci is 4.3 *N*_e_ *m* into the CA from WP and 3.2 *N*_e_ *m* from CA into WP. Because the 95% CI do not overlap WP is listed as the source.

Parameter estimates are listed for each cluster pair^*^, as well as the directionality of gene flow between cluster pairs (source, sink, and symmetrical [–]) assigned on the basis of 95% confidence intervals (in parentheses). The Eastern Polar Basin cluster (EP) includes polar bears from East Greenland, Barents Sea, Kara Sea and Laptev Sea subpopulations. The Western Polar Basin cluster (WP) includes polar bears from the Chukchi Sea, southern Beaufort Sea and northern Beaufort Sea. The Canadian Archipelago cluster (CA) includes Viscount Melville, M'Clintock Channel, Gulf of Boothia, Lancaster Sound, Norwegian Bay, Kane Basin, Baffin Bay and the region north of Hudson Strait in Davis Strait (DS). The Southern Canada cluster (SC) includes Foxe Basin, Southern Hudson Bay, Western Hudson Bay and the region south of Hudson Strait in DS.

Among gene flow estimates based on mtDNA, there was a signal of effective dispersal from the Southern Canada cluster into both Eastern and Western Polar Basin clusters (Table 1).

### Fluctuations in Historical Effective Population Size

Globally, using mtDNA, polar bears showed a strong signal of historical population growth (>18 times the SD; *g* = 915.33, SD = 48.01), and the mismatch distribution did not deviate significantly from a sudden expansion model (SSD = 0.044, *P* = 0.103) but did show significant raggedness (rg = 0.013; S1 Table). Based on the coalescent, the Western and Eastern Polar Basin and Southern Canada clusters showed a significant signal of historical growth (S1 Table). However, significant geographic expansion (and growth) is consistently evident for only a few subpopulations within these regions: for the Eastern Polar Basin, only the LP subpopulation; for the Western Polar Basin, the CS and SB; and for Southern Canada, only SH showed significant historical expansion (S1 Table). CS and SH each exhibited a particularly strong signal of growth (>6, and>19 times SD, respectively; S1 Table); both subpopulations were also characterized by significantly negative Fu's *Fs* and, for SH, also a significantly negative Tajima's *D* (S1 Table). Among subpopulations with strong growth signals, mismatch distributions for the LV, SB and CS did not differ significantly from a sudden expansion model, although the latter showed a signal of significant raggedness (S1 Table). Similarly, mismatch distributions for SH did not deviate from a sudden expansion model (S1 Table). The Canadian Archipelago cluster did not show a signal of growth overall, nor did its component subpopulations (S1 Table). Extended Bayesian plots did not indicate significant growth within regional clusters (S5b-e Fig.), although a signature of growth was observed overall (S5a Fig.).

We estimated time since population expansion, based on mismatch distributions (S6 Fig.), a mutation rate of 11.0% per million years (based on our coalescent Bayesian analysis of mtDNA that was internally calibrated by incorporating data from the entire mitogenome of a 120,000 year old polar bear fossil [36]), and based on a generation time of ten years for polar bears and six years for brown bears (following [33]), to be approximately 98,260 (τ = 2.512) and 56,410 (τ = 1.405) years ago, respectively, for polar bears of the Western Polar Basin cluster (SB and CS, pooled) and brown bears of the Eastern Beringian Clade. Since the algorithm assumes population expansion, the proposed expansion date for the Western Beringian brown bear clade (31,079 years, τ = 1.290) is tentative (S9 Table). We note that a lineage-specific mutation rate of 11% for the mtDNA control region is lower than the 30% multiple calibration rate estimated by Saarma et al. [79] for tip rates, but greater than their 5% root estimate.

### Phylogenetic relationships

As observed in previous studies based on mtDNA gene sequences [35], [37], extant brown bears are paraphyletic with respect to polar bears (Fig. 1a); brown bears from Admiralty, Chichagof and Baranof (ABC) islands of the Alexander Archipelago of southeastern Alaska share a mtDNA lineage more similar to polar bear than other brown bears in North America, due to recent [41] or ancient [33], [36] hybridization, incomplete lineage sorting [37], [80], or both. Further, in contrast to brown bears of eastern and western Beringia, modern polar bears show relatively shallow within-species divergence; control region haplotypes in brown bears of eastern Beringia differ from western Beringian haplotypes by at least 3.0%, whereas polar bear haplotypes differ by less than 0.5% (Fig. 1a,b). The results of minimum evolution analyses were largely congruent with analyses based on maximum parsimony and maximum likelihood. All analyses placed homologous sequence data generated from a ca. 120 ka polar bear fossil from Poolepynten (Svalbard) see [33] phylogenetically closer to the ABC brown bear clade relative to the majority of polar bear haplotypes globally (Fig. 1a). Average corrected genetic distances between modern polar bear haplotypes and haplotypes found in ABC brown bears were more than twice as large as average within-polar and within-ABC brown bear haplotype distances (S10 Table). The average corrected distance between the modern polar bear group and the Poolepynten polar bear (TrN+I+G = 0.012) was the same as the distance between the latter and the ABC brown bears (S10 Table).

## Discussion

### Contemporary response to changing climate

We detected an increase in directional gene flow of polar bears from the Eastern Polar Basin towards the Canadian Archipelago and Western Polar Basin, and from Southern Canada to the Canadian Archipelago, within the last 1–3 generations (bayesass analysis of microsatellite DNA). The directional gene flow from the Eastern Polar Basin to the Canadian Archipelago and Western Polar Basin is specifically contemporary (past 1–3 generations; bayesass analyses of microsatellite DNA), occurring during this current era of sea-ice decline. Estimates of asymmetry in historical gene flow did not show a similar pattern: coalescent estimates of asymmetry in gene flow (migrate analyses) did not show directionality based on maternally-inherited mtDNA (late Pleistocene signature), or on microsatellite DNA (Holocene signature). The northern parts of the Canadian Archipelago have been predicted to retain polar bear ice habitat farther into the future than other Arctic areas [6], [17]. This directional gene flow from the Eastern Polar Basin cluster towards the Canadian Archipelago and Western Polar Basin clusters is supported by predominant seasonal ice movement from the Eastern Polar Basin (e.g., Laptev Sea) towards the northern edge of the Canadian Archipelago and northern Greenland i.e., part of the Beaufort Gyre [81]. As the extent of sea-ice continues to decrease in response to climate change, the northern edge of the Canadian Archipelago and adjacent areas of the Polar Basin are predicted to retain the last vestiges of summer and autumn sea-ice habitat [6], [17]. Our findings constitute empirical evidence in support of this hypothesis. Note although the Beaufort Gyre moves ice from the western portion of the Western Polar Basin towards the Canadian Archipelago, we did not detect gene flow in this particular direction.

Throughout the Holocene, polar bears in the Canadian Archipelago have had year-round access to sea-ice from which to hunt for seals. Conversely, in much of the seasonal-ice ecoregion [6] in the eastern Arctic and subarctic of Canada (i.e., our Southern Canada cluster), polar bears spend several months fasting ashore. The climate warming of recent decades [1] has led to earlier break-up of sea ice and longer ice-free seasons in these subpopulations of the Southern Canada cluster. Decreased body condition, lower survival, and reduced abundance of polar bears in some of subpopulations in the Southern Canada cluster have been linked to these sea ice changes [9], [11], [13], but see [15]). In contrast, some researchers hypothesize the Canadian Archipelago will become more productive habitat for polar bears as annual ice over shallow waters (better conditions for seal prey) will replace thick, multi-year and less productive ice habitat [5]. Derocher et al. [19] and Durner et al. [17] predicted such a northerly shift in polar bears as productive habitat changes as a result of climate warming. Our empirical gene flow data support this prediction. In addition to patterns of ice movement within the Polar Basin, and changes in availability of preferred polar bear habitat, evidence of net gene flow into the Canadian Archipelago and Western Polar Basin may also be influenced by asymmetries in harvest pressure. The Canadian Archipelago and the Western Polar Basin have at least 2.8 times and 1.5 times, respectively, higher polar bear harvest than in the Eastern Polar Basin [22] in which only the East Greenland subpopulation is harvested.

### Contemporary Genetic Structure

Our examination of asymmetry in gene flow was based on results of Bayesian clustering which grouped the circumpolar population of polar bears into three or four regional genetic clusters that represent patterns of breeding within the recent Holocene (Fig. 2a,b). Generally, individual polar bears from entire subpopulations were assigned to one of three regional clusters. The exception was the Davis Strait subpopulation, which was split between two regional clusters: Southern Canada and the Canadian Archipelago. Targeted Bayesian analyses also supported the further division of the Polar Basin cluster into the Eastern and Western Polar Basins (S3c Fig.). Although three regional groupings were also uncovered using traditional metrics (AMOVA analyses of both microsatellite loci and mtDNA), only mtDNA AMOVA analyses supported the presence of the four regional clusters uncovered using Bayesian clustering, which includes the eastern and western Polar Basin split (S7 Table). The greater level of population and regional structuring uncovered using data from the maternally-inherited mtDNA relative to the biparentally-inherited nuclear microsatellite loci is not surprising. Pairwise *F*_ST_ values for microsatellite loci are generally lower than expected based on *F*_ST_ values for mtDNA, reflecting hypothesized greater levels of philopatry among females (S4 Fig.).

Oceanographic patterns of ice formation, ice melt, and the convergence and divergence of ice from shore create different ecological circumstances for polar bears [6] that likely contribute to these clustering patterns. The Canadian Archipelago cluster is confined by narrow, shallow straits between islands, insulating the region from the ice dynamics of the Polar Basin. The Canadian Archipelago was identified by Amstrup et al. [6] as one of four unique eco-regions for polar bears. Three subpopulations – Kane Basin, Baffin Bay and the more northerly region of Davis Strait – group with the subpopulations in the Canadian Archipelago proper (Lancaster Sound, Norwegian Bay, M'Clintock Channel, Viscount Melville, Gulf of Boothia) into the Canadian Archipelago genetic cluster. This is consistent with a general northerly seasonal ice movement and the directional gene flow towards the Canadian Archipelago. The grouping of these additional subpopulations with the Canadian Archipelago proper supports the proposed “Central Arctic” conservation unit of polar bears defined based on ecological similarities [48].

The Southern Canada cluster includes subpopulations in the Hudson Bay ecological complex [48], [82], for which breeding habitat overlaps in central Hudson Bay [21]. Polar bears sampled south of Hudson Strait in the Davis Strait subpopulation also clustered with the Hudson Bay complex of polar bears. The marked genetic differences between southern and northern Davis Strait bears must reflect differences in southern versus northern breeding areas in the spring. However, polar bears in Davis Strait were largely sampled in the fall; this suggests that the fidelity to the two subregions within Davis Strait is year-round. Polar bears occupying southern and northern Davis Strait also differ in diet [83] and rates of survival and recruitment [15]. Though movement has been documented between these two regions [24], [84], [85], movement across Hudson Strait may be seasonal or has not resulted in significant evolutionary dispersal. As Hudson Strait becomes ice-free ever-earlier in spring [86], with continued climate warming [20], the division between northern and southern Davis Strait may increase. Also within the Southern Canada cluster, we found similar genetic substructure, albeit shallow, within the Southern Hudson Bay subpopulation, confirming significant divergence between polar bears sampled during the autumn on the Ontario Coast of Hudson Bay and those sampled on Akimiski Island within James Bay [21]. The restricted genetic interchange within both Davis Strait and Southern Hudson Bay exacerbates the conservation concern of these two populations, which are at the southern fringe of current polar bear distribution [15], [87], [88].

Increasingly restricted gene flow as ice extent continues to decline could drive the current system, which appears to be moderately structured among broad regions but less structured among subpopulations, toward one characterized by meta-population dynamics. If periodic immigration into increasingly isolated subpopulations were not possible, subpopulations could become increasingly vulnerable to extirpation. Yet, our failure to detect shifts in distributions of alleles within the nine subpopulations of polar bears that were sampled over the past three decades (i.e., 1980s-early 2010s), indicate that demographic fluctuations during the past several generations have been insufficient to influence genetic partitioning of subpopulations within regions.

### Historical Response to Changing Climates

Mismatches between expansions and contractions of polar bears and brown bears appear to coincide with past climate fluctuations. We timed the expansion of Western Polar Basin polar bears to the early Wisconsin glacial period (approximately 98,000 years ago), preceding the expansion of Western Beringian brown bears before the Last Glacial Maximum (LGM), approximately 31,000 years ago, and Eastern Beringian brown bears during the last glacial at ∼56,000 years ago. Deep nuclear and mitogenomic sequencing (of a few individuals) attributed to differential responses to climate changes and hybridization [33], [40], [41] corroborate out of phase population trends. Deep nuclear genomic sequencing suggested that brown bear effective population size apparently increased during the last interglacial (approximately 130–114 kya), and decreased during the last glacial period. The opposite pattern was uncovered for polar bears, which showed a marked increase in population size coincident with Pleistocene cooling, and a decrease coincident with climate warming associated with the last interglacial [33]. Signatures of evolutionarily recent events (i.e., subsequent to the LGM) may have low power from whole genomic data [33]. However, similar to other studies [36], our analyses, based on multiple expansion and growth metrics, suggest that polar bear population sizes have fluctuated in response to climate cycling. Lack of consistency across these metrics, however, may benefit by simulated tests of directed hypotheses that investigate timing and extent of effective population size changes [89], [90], and account for among-lineage variation in comparative demographic analyses [91].

The phylogenetic relationships and timing of lineage divergence within and between polar and brown bears remain controversial, due to the difficulty in discerning between incomplete lineage sorting and ancient and recent (periodic) hybridization between the two species [41], [92], [93], coupled with uncertainty in estimates of marker-specific [92] and genome-wide mutation rates [90] when comparing across species. Deep nuclear genomic sequence data places the initial divergence between ancestors of modern polar bears and brown bears much earlier (ca. 4 mya; [33], although see [90], than estimates based on single nucleotide polymorphisms (ca. 1.2 mya; [94]), 14 nuclear introns (ca. 0.23–0.93 mya; [95], 13 Y chromosome markers (1.12 mya; [96]), or on the mitochondrial genome alone (ca. 150,000 years ago; [36]). The latter estimate may reflect the capture of the polar bear mitogenome by brown bears, due to a hybridization event, rather than the initial divergence of the species. Analysis of mitogenomic data, including data from an ancient (120,000 year old) fossil polar bear, suggests an age for the extant polar bear matrilineage of less than 45,000 years, consistent with recent and rapid growth of modern polar bear populations toward the end of the last Pleistocene glacial but prior to the LGM [33]. The shallow within-species structure of modern polar bears relative to brown bears, reflected in our mtDNA haplotype tree (based on control region sequences from over 400 polar bears), supports the hypothesis that modern polar bears stem from a single refugial lineage. Fossil DNA evidence [33] indicates potential loss of diversity due to extirpated lineages. Thus, as with other species [97]–[99] analyses of ancient DNA sequences in polar bears has provided insight into evolutionarily recent events by facilitating the calibration of molecular clocks and detecting signals of past population shifts.

### Interglacial Refugia and Hybridization

The Svalbard region has been proposed as a previous interglacial refugium, retaining a source population for range expansion during cooler (glacial) periods [33]. We observed a signal of stability among polar bears comprising the Canadian Archipelago and the Barents Sea subpopulations (i.e., Svalbard) within the Eastern Polar Basin. This indicates that these areas may have served as previous interglacial refugia and provided leading-edge expansion of polar bears into other areas during glacial periods. Combined with our evidence of contemporary gene flow, this supports the hypothesis that these regions may become a future refugium for polar bears. Although analyses of Y chromosome loci failed to uncover introgression of the polar bear Y chromosome into brown bears and vice versa [96], analyses of both the nuclear autosomal and the mitochondrial genome suggest that brown and polar bears possess introgressed alleles [95], [33], [41]. Recently, analysis of mtDNA extracted from fossil bear samples from two proposed interglacial refugia – one in northern Europe (Ireland; [40]), and one in the Alexander Archipelago in Alaska [41] – indicates that modern polar bears stem from one or several hybridization event(s) between polar bears and brown bears co-occupying periglacial late Pleistocene habitats. In northern Europe, hybridization is timed to approximately 28–32 kya [40] and in southeast Alaska is inferred to be between 26 kya years ago and the Pleistocene/Holocene boundary [41]. Analysis of entire mitogenomes from brown and polar bears [33], however, places their common matrilineal ancestor (from the Alexander Archipelago) at ca. 152 kya, with radiation of the modern polar bear mitochondrial crown group at ca. 44 kya. Any hybridization event must therefore have occurred within this interval, substantially predating the hybridization event proposed by Cahill et al. [41]. Our mtDNA control region data are also inconsistent with a recent hybrid origin of modern polar bears, at least in the proposed Alexander Archipelago refugium (92 kya for coalescence of polar bears and Alexander Archipelago brown bears; 64 kya for coalescence of the polar bear crown group; S10 Table), and suggest an earlier (pre-LGM) hybridization event. Similar to data from the Y chromosome [96] and earlier analyses of a reduced suite of microsatellite loci and smaller geographic coverage [45], we found no evidence of contemporary admixture between polar bears and brown bears. Though F1 and F2 hybrids have recently been observed in the Northern Beaufort Sea and Viscount Melville [42], our extensive sampling suggests this current hybridization is thus far localized.

### Sex Bias in Gene Flow

It is not possible to place radio collars on male polar bears due to the males' neck circumferences exceeding the circumference of the skull, and tracking male polar bears by other means has had mixed success. Limited data have revealed no differences between the male and female movement distances (but see [24], [100], [101]). In contrast to other bear species, it is evident that female polar bears, even with young, cover extremely large distances on their mobile ice platforms [24]. In contrast, we also know that movements of female polar bears with young of the year are often restricted during the spring [102] and seasonal fidelity can be high [103]. Also in contrast to other ursids, movements for both sexes do not appear to be constrained due to territorial behavior. Slight male-biased dispersal and gene flow, however, was found using molecular techniques within the Barents Sea subpopulation [28], suggesting that sex-specific movement patterns, resulting in gene flow, do occur. Our joint analysis of biparentally-inherited and maternally-inherited genomes across 18 subpopulations suggest that gene flow is mediated more by male polar bears, and that females show higher natal fidelity.

### Future Research Direction

Though we increased the geographic sampling of polar bears, our study was limited by lack of modern samples from the Kara Sea in the Russian Arctic (Eastern Polar Basin cluster). This region is of conservation concern due to hydrocarbon exploration and unquantified levels of poaching [8], and should be a target of research, including research on genetic diversity. New samples from the Northern Beaufort Sea, and a reanalysis for asymmetry in gene flow within the Western Polar Basin and towards the Canadian Archipelago could test our finding of gene flow towards this region. We predict gene flow into this subpopulation because of declining polar bear individual and population status in the Southern Beaufort Sea [10], [12], the direction of annual ice drift towards the Northern Beaufort Sea, and the stability or slight increase in abundance of bears in the Northern Beaufort Sea [104]. In addition, because our data suggest gene flow into the Canadian Archipelago, and because this region is likely to retain ice habitat longer into the future [6], [17] than other parts of the Arctic, new sampling should focus on Norwegian Bay, Nunavut, Canada, for which we did not obtain new samples, and the Queen Elizabeth Islands, which are specifically in the convergent ice zone of the Canadian Archipelago [6]. Further, analyses of additional samples from the Northern Beaufort Sea and neighboring Viscount Melville subpopulations may yield signals of more contemporary hybridization events [42]. Finally, with the isolation of functional single amino acid polymorphisms for polar bears [33], comparing functional and neutral genetic diversity with variation in ecological strategies [6] should prove informative for understanding adaptation to past, current and future environments.

### Conservation Implications

Our work updates and expands previous circumpolar genetic analyses of polar bears [27], [45], corroborating and refining those results. We exposed the asymmetries in gene flow among subpopulations, and also provided a deeper historical context by combining analyses of microsatellite and mtDNA data. The signal of novel and recent gene flow towards the Canadian Archipelago, a potential refugium [8], supports increasing research, monitoring and proactive conservation in this region. The relatively high genetic diversity we report for polar bears provides an expanded baseline for future comparisons as climate change and harvest continue to impact polar bear distribution, connectivity and genetic diversity [20]. Given that Arctic habitats are changing rapidly, our analyses provide evidence of potential future centers of diversity for polar bears, and insight into differential responses of polar and brown bears (sister species) to common environmental processes. Our work provides a circumpolar perspective on how changing habitat is influencing gene flow in a species of worldwide conservation concern, and illustrates the value of incorporating genetic information in analyses to understand the response of species to climate change.

## Supporting Information

S1 Fig **Locations of polar bears, a., sampled at known latitude and longitude (n = 2,650) in 18 circumpolar subpopulations of polar bears, recognized by the IUCN/Polar Bear Specialist Group, and amplified at microsatellite loci: Baffin Bay (BB); Barents Sea (BS); Chukchi Sea (CS); Davis Strait (DS); East Greenland (EG); Foxe Basin (FB); Gulf of Boothia (GB); Kane Basin (KB); Kara Sea (KS); Laptev Sea (LP); Lancaster Sound (LS); M'Clintock Channel (MC); Northern Beaufort Sea (NB); Norwegian Bay (NW); Southern Beaufort Sea (SB); Southern Hudson Bay (SH); Viscount Melville (VM); and Western Hudson Bay (WH).** Circles identify bears sampled at known latitude and longitude in the 1980s (n = 157), 1990s (n = 613), 2000s (n = 1,708) and 2010s (n = 183). **b**. Locations of 402 polar bears samples in 15 subpopulations with known latitude and longitude amplified at the mitochondrial DNA control region.
(TIFF)

S2 Fig **The average (95% Confidence Intervals) of 5 runs per cluster, of the negative log likelihood of the probability of the microsatellite data given the number of clusters of polar bears, K, simulated by the program structure (1), in the circumpolar Arctic.**
(TIFF)

S3 Fig **Genetic substructure of polar bears within the three broad clusters identified by program structure (1): a. two most likely sub-clusters within the Canadian Archipelago Cluster [(Viscount Melville (VM), Gulf of Boothia (GB), Norwegian Bay (NW), Lancaster Sound (LS), Kane Basin (KB)] with Baffin Bay (BB) and Davis Strait (DS;) b. two most likely sub-clusters within the Southern Canada Cluster [(Western Hudson Bay (WH), Southern Hudson Bay (SH) and Foxe Basin (FB)) and Davis Strait (DS; sorted by high to low latitude)] and c. the two most likely sub-clusters within the Polar Basin: the Eastern Polar Basin Cluster [(East Greenland (EG), Barents Sea (BS), Kara Sea (KS), Laptev Sea (LS)] and the Western Polar Basin Cluster [Chukchi Sea (CS), Southern Beaufort Sea (SB) and Northern Beaufort Sea (NB)].** All individuals are sorted left to right by high to low latitude.
(TIF)

S4 Fig **Scatter plot of observed pairwise mtDNA Φ_ST_ versus pairwise microsatellite** ***F*** **_ST_ values for 21 microsatellite loci (circles) for 15 subpopulations of polar bears.** The line represents the expected microsatellite *F*_ST_ value given the genetic differentiation observed at mtDNA (2): *F*_ST_(nu) = 1–*e* ^0.25*ln[1–*F*ST(mt)]^. Generally the pairwise comparisons are below the expectation line (i.e., lower *F_ST_* derived from microsatellite markers compared with the mtDNA), which suggests higher female philopatry relative to males (i.e., male biased gene flow). Stars show similar comparisons of pairwise mtDNA Φ_ST_ and microsatellite *F*_ST_ values for brown bear populations in Alaska. Black circles show values that represent polar bear subpopulations that are ≤900 kilometers between each other for comparison to the brown bear populations shown, which are 900 km apart from each other.
(TIFF)

S5 Fig **Extended Bayesian skyline plots (EBSPs) and pairwise mismatch distributions for (a) global-wide (b) Eastern Polar Basin, (c) Western Polar Basin, (d) Canadian Archipelago, and (e) Southern Canada clusters of polar bears.** EBSPs indicate population growth from past (right) to present (left) including median population size through time (black line) and 95% highest probability distribution (grey interval). Log-transformed *y*-axes represent population size as a function of effective size (*N*_e_) and generation time (*G*). Mismatch distributions indicate the frequency of expected (grey line) and observed (black) pairwise differences.
(TIFF)

S6 Fig **Mismatch distributions of pairwise differences based on mtDNA data of polar bears from the southern Beaufort (SB) and Chukchi Sea (CS) subpopulations and brown bears from the Western and Eastern Beringian Clades.** Mismatch signals between the lineages are offset, signifying different periods of demographic growth.
(TIF)

S1 Table **Estimates of genetic diversity in 18 circumpolar subpopulation of polar bears, arranged within the four genetic clusters identified in this paper, including allelic richness (AR), observed heterozygosity (H_o_), expected heterozygosity (H_e_) for the microsatellite data; and the number of haplotypes (** ***k*** **), haplotype (** ***h*** **) and nucleotide diversity (** ***π*** **), and Ewen-Wattersen's neutrality (E** ***ν*** **) for the mitochondrial DNA control region.** Genetic demographic statistics include the growth statistic (*g*) with standard deviation (bold if significant where *g*≥SD[*g*]), Fu's *Fs*, Tajima's *D*, raggedness (*rg*), and Deviation from a sudden expansion (SSD). Bold values for microsatellite data signify microsatellite data from subpopulations that were out of Hardy-Weinberg Equilibrium at α = 0.05/number of loci. Bold values for demographic statistics indicate significance at *P*≤0.05. Abbreviations of subpopulations are as follows: Baffin Bay (BB); Barents Sea (BS); Chukchi Sea (CS); Davis Strait (DS); East Greenland (EG); Foxe Basin (FB); Gulf of Boothia (GB); Kane Basin (KB); Kara Sea (KS); Laptev Sea (LP); Lancaster Sound (LS); M'Clintock Channel (MC); Northern Beaufort Sea (NB); Norwegian Bay (NW); Southern Beaufort Sea (SB); Southern Hudson Bay (SH); Viscount Melville (VM); and Western Hudson Bay (WH).
(DOCX)

S2 Table **Distribution of haplotypes (and GenBank accession numbers) at the mitochondrial DNA control region in 18 subpopulations of polar bears: Baffin Bay (BB); Barents Sea (BS); Chukchi Sea (CS); Davis Strait (DS); East Greenland (EG); Foxe Basin (FB); Gulf of Boothia (GB); Kane Basin (KB); Kara Sea (KS); Laptev Sea (LP); Lancaster Sound (LS); M'Clintock Channel (MC); Northern Beaufort Sea (NB); Norwegian Bay (NW); Southern Beaufort Sea (SB); Southern Hudson Bay (SH); Viscount Melville (VM); Western Hudson Bay (WH).**
(DOCX)

S3 Table **Genetic differentiation results of comparisons of microsatellite data from earlier and later samples within nine global subpopulations and regions of polar bears: the Svalbard portion of the Barents Sea (BS); Baffin Bay (BB); Chukchi Sea (CS); Foxe Basin (FB); Gulf of Boothia (GB); the Labrador portion of Davis Strait (DS); Lancaster Sound (LS); Southern Beaufort Sea (SB) and Western Hudson Bay (WH).** Degrees of freedom are shown in parentheses. The K metric represents the likely number of clusters for the subpopulation or region with decadal data pooled as ascertained using the Bayesian clustering program baps. Values in bold show significant differentiation between the groups (α = 0.05, Bonferroni corrections applied).
(DOCX)

S4 Table **Below the diagonal, significant (+) and non-significant (−) differences for genic differentiation, F_ST_, and genotypic differentiation (the latter only for comparisons of subpopulations that were found to be out of Hardy-Weinberg equilibrium) for pairwise comparisons of 18 subpopulations of polar bears using microsatellite data.** Bonferroni-corrected significance levels were adjusted for the number of loci compared for each pair (0.05/number of loci compared). Shaded blocks delineate the four clusters based on our analysis: Eastern Polar Basin, Western Polar Basin, Canadian Archipelago and Southern Canada. Above the diagonal: significant (+) and non-significant (−) differences for F_ST_, θ_ST_ and haplotypic differentiation using mtDNA data for pairwise comparisons of 15 subpopulations. Abbreviations for subpopulations are as follows: Baffin Bay (BB); Barents Sea (BS); Chukchi Sea (CS); Davis Strait (DS); East Greenland (EG); Foxe Basin (FB); Gulf of Boothia (GB); Kane Basin (KB); Kara Sea (KS); Laptev Sea (LP); Lancaster Sound (LS); M'Clintock Channel (MC); Northern Beaufort Sea (NB); Norwegian Bay (NW); Southern Beaufort Sea (SB); Southern Hudson Bay (SH); Viscount Melville (VM); and Western Hudson Bay (WH).
(DOCX)

S5 Table **Pairwise estimates of population differentiation among 15 (mitochondrial DNA) or 18 (microsatellite DNA) circumpolar subpopulations of polar bears: Baffin Bay (BB); Barents Sea (BS); Chukchi Sea (CS); Davis Strait (DS); East Greenland (EG); Foxe Basin (FB); Gulf of Boothia (GB); Kane Basin (KB); Kara Sea (KS); Laptev Sea (LP); Lancaster Sound (LS); M'Clintock Channel (MC); Northern Beaufort Sea (NB); Norwegian Bay (NW); Southern Beaufort Sea (SB); Southern Hudson Bay (SH); Viscount Melville (VM); Western Hudson Bay (WH).** Significant values (α = 0.002 and 0.05 for microsatellite and mtDNA comparisons, respectively) are in bold text.
(DOCX)

S6 Table **Genetic differentiation within the Southern Hudson Bay (SH) and Davis Strait (DS) subpopulations of polar bears sampled during autumn while bears are on land.** In the two-way comparison within DS: Northern indicates samples from polar bears north of Hudson Strait; Southern indicates samples south of Hudson Strait; samples from the Hudson Strait portion of the Foxe Basin subpopulation are not included in this comparison. In the three-way comparison: Southern, as above; Northern Baffin includes samples north of Frobisher Bay on Baffin Island to the border with the Baffin Bay subpopulation; Hudson Strait includes samples south of Frobisher Bay on Baffin Island and along Hudson Strait, including adjacent Hudson Strait samples from the Foxe Basin subpopulation. Akimiski Island is in James Bay, which is in the SH subpopulation; the Hudson Bay Coast is also within SH. All values are significant at α = 0.05/number of loci.
(DOCX)

S7 Table **Hierarchical analysis molecular variance (AMOVA) among subpopulations and various groupings of microsatellite (θ_CT_, θ_SC_) and mitochondrial (Φ_CT_,Φ_SC_) alleles to test hypotheses of groupings of the global subpopulations of polar bears: Baffin Bay (BB); Barents Sea (BS); Chukchi Sea (CS); Davis Strait (DS); East Greenland (EG); Foxe Basin (FB); Gulf of Boothia (GB); Kane Basin (KB); Kara Sea (KS); Laptev Sea (LP); Lancaster Sound (LS); M'Clintock Channel (MC); Northern Beaufort Sea (NB); Norwegian Bay (NW); Southern Beaufort Sea (SB); Southern Hudson Bay (SH); Viscount Melville (VM); and Western Hudson Bay (WH).** Bold values are significant at α = 0.05/number of loci  = 0.0023–0.003 (for microsatellite data) or 0.05 (for mtDNA data).
(DOCX)

S8 Table **The proportion of non-migrants (95% CI) over the last ca. 1–3 generations for four genetic clusters of polar bears calculated using program bayesass (1).** The Eastern Polar Basin Cluster includes polar bears from East Greenland, Barents Sea, Kara Sea and Laptev Sea subpopulations. The Western Polar Basin Cluster includes polar bears from the Chukchi Sea, Southern Beaufort Sea and Northern Beaufort Sea subpopulations. The Canadian Archipelago Cluster includes Viscount Melville, M'Clintock Channel, Gulf of Boothia, Lancaster Sound, Norwegian Bay, Kane Basin, Baffin Bay MUs, and the region north of Hudson Strait in the Davis Strait subpopulations. The Southern Canada Cluster includes Foxe Basin, Southern Hudson Bay, Western Hudson Bay MUs, and the region south of Hudson Bay in the Davis Strait subpopulations. Theta (θ) for each cluster is calculated from microsatellite (effective population size (N_e_), scaled to mutation rate (µ) and mtDNA data (female effective population size (N_f_), scaled to mutation rate) using the program MIGRATE.
(DOCX)

S9 Table **Coalescent times (thousands of years ago) to most recent common ancestor for major mitochondrial lineages of brown and polar bears based on 581 bp of the mitochondrial DNA control region, excluding indels.** Provided are nodal support for lineages based on Bayesian analysis in BEAST and median age of nodes with 95% confidence intervals (CI). ABC bears refer to brown bears from the Alexander Archipelago, Alaska, USA.
(DOCX)

S10 Table Average pairwise distances within and among haplotypes from polar bears sampled from 15 subpopulations, the ancient Poolypenten (GenBank Accession No. GU573488)* polar bear and haplotypes found within the three clades of Alaskan brown bears. Values were generated using the Tamura-Nei (I+G_0.69_) model of substitution.
(DOCX)

S11 Table **Metadata associated with samples of polar bears collected for microsatellite and mitochondrial DNA (mtDNA) analysis.** *Table in MS Excel Format*.
(XLSX)

S12 Table **Permissions and permits for collection of tissue samples of polar bears used in this study.**
(DOCX)

S1 Supporting Information **Detailed materials and methods.**
(DOCX)
