# Correction: Implications of the circumpolar genetic structure of polar bears for their conservation in a rapidly warming Arctic

**DOI:** 10.1371/journal.pone.0136126

**Published:** 2015-08-14

**Authors:** Elizabeth Peacock, Sarah A. Sonsthagen, Martyn E. Obbard, Andrei Boltunov, Eric V. Regehr, Nikita Ovsyanikov, Jon Aars, Stephen N. Atkinson, George K. Sage, Andrew G. Hope, Eve Zeyl, Lutz Bachmann, Dorothee Ehrich, Kim T. Scribner, Steven C. Amstrup, Stanislav Belikov, Erik W. Born, Andrew E. Derocher, Ian Stirling, Mitchell K. Taylor, Øystein Wiig, David Paetkau, Sandra L. Talbo

The following sentence was omitted and should have been included as the last sentence of the first paragraph in the Estimation of Gene Flow section of S1 Supporting Information:

Because we wanted input dataset for the gene flow analyses of microsatellite data to 1) be comparable for both MIGRATE and BayesAss analyses; 2) avoid as much as possible the use of individuals with large amounts of missing data; and because 3) 15 individuals are recommended (for efficiency) for the MIGRATE analyses (P. Beerli and M. Kuhner, pers. comm. to SAS), and we wanted each area to be fairly equally represented, we randomly sampled individuals from strata (geographic areas) within each subpopulation, selecting from 26 (WP), 34 (EP) and 60 (CA and SC), depending on the number available for analysis.

Please see the correct, complete S1 Supporting Information here.

## Supporting Information

S1 Supporting Information Detailed materials and methods. (DOCX)
