# Supplementary figures and images for: Implications of the Circumpolar Genetic Structure of Polar Bears for Their Conservation in a Rapidly Warming Arctic
Source: PLoS One. 2015 Jan 6;10(1):e112021. doi: 10.1371/journal.pone.0112021 (PMC4285400; doi:10.1371/journal.pone.0112021)

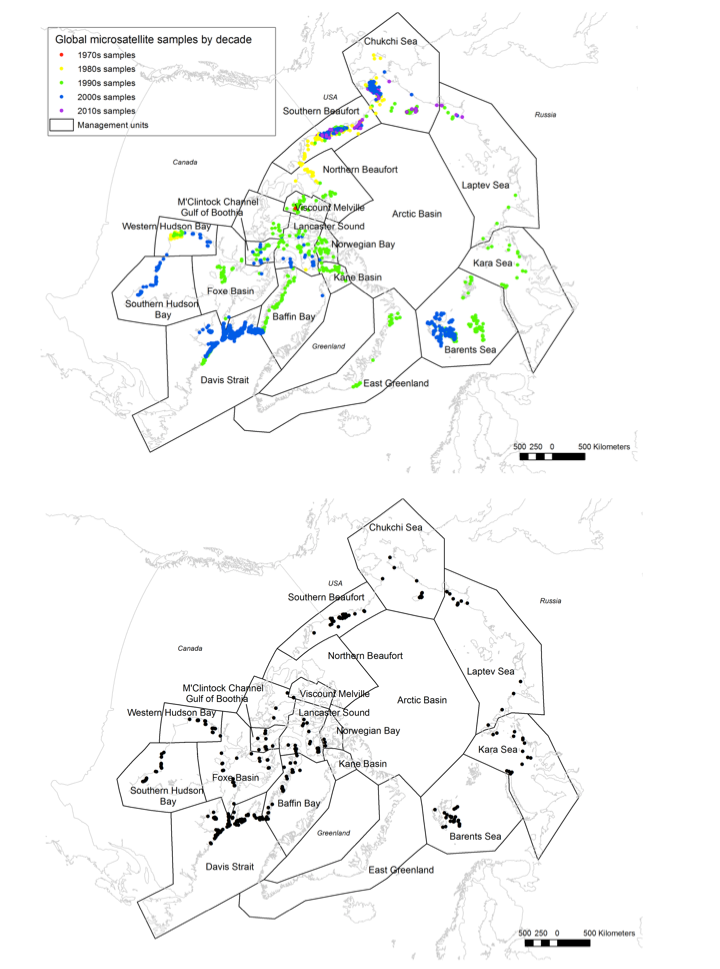

Supplement: S1 Fig — Locations of polar bears, a., sampled at known latitude and longitude (n = 2,650) in 18 circumpolar subpopulations of polar bears, recognized by the IUCN/Polar Bear Specialist Group, and amplified at microsatellite loci: Baffin Bay (BB); Barents Sea (BS); Chukchi Sea (CS); Davis Strait (DS); East Greenland (EG); Foxe Basin (FB); Gulf of Boothia (GB); Kane Basin (KB); Kara Sea (KS); Laptev Sea (LP); Lancaster Sound (LS); M'Clintock Channel (MC); Northern Beaufort Sea (NB); Norwegian Bay (NW); Southern Beaufort Sea (SB); Southern Hudson Bay (SH); Viscount Melville (VM); and Western Hudson Bay (WH). Circles identify bears sampled at known latitude and longitude in the 1980s (n = 157), 1990s (n = 613), 2000s (n = 1,708) and 2010s (n = 183). b. Locations of 402 polar bears samples in 15 subpopulations with known latitude and longitude amplified at the mitochondrial DNA control region. (TIFF) [file pone.0112021.s001.tiff]

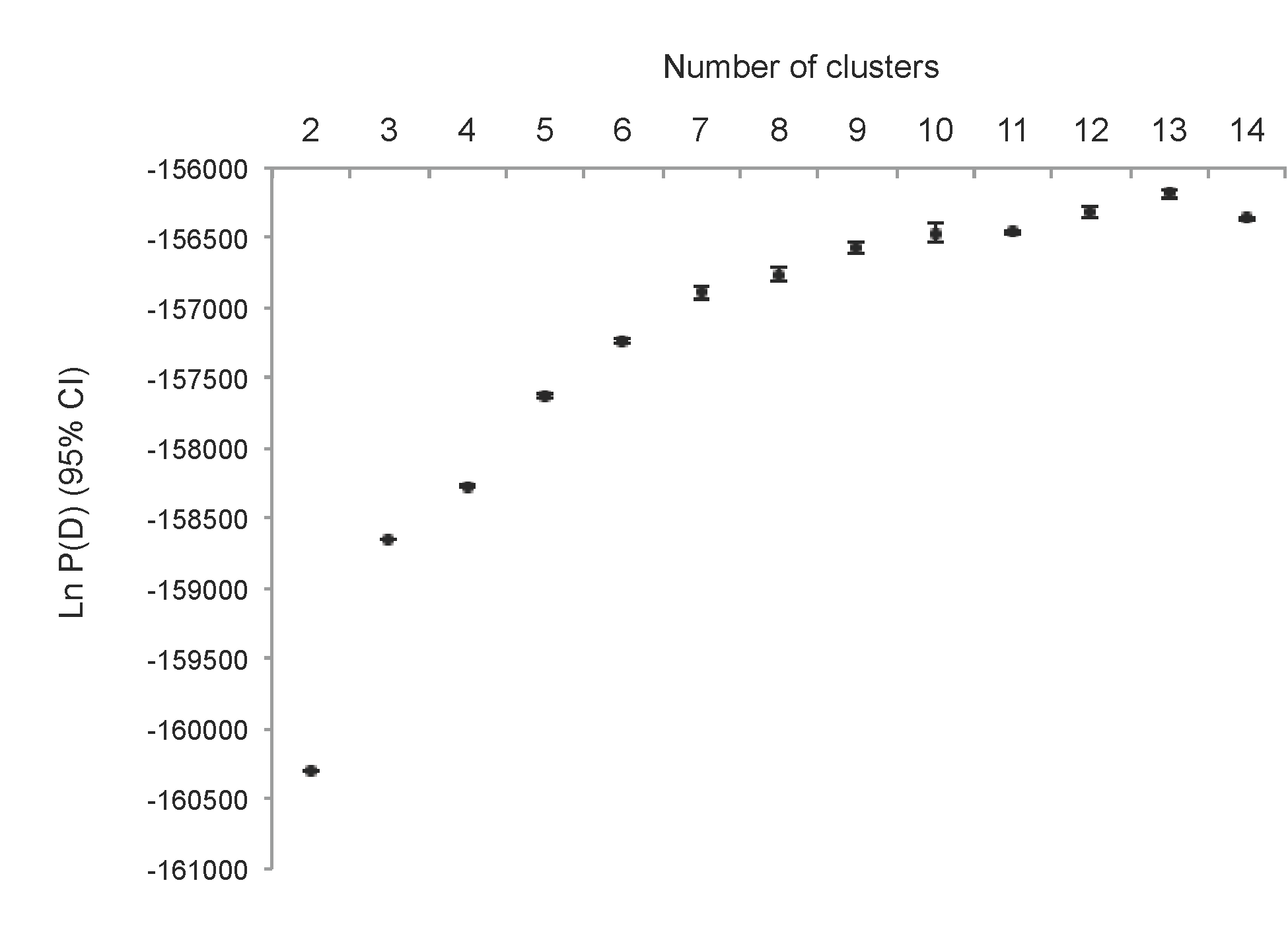

Supplement: S2 Fig — The average (95% Confidence Intervals) of 5 runs per cluster, of the negative log likelihood of the probability of the microsatellite data given the number of clusters of polar bears, K, simulated by the program structure (1), in the circumpolar Arctic. (TIFF) [file pone.0112021.s002.tiff]

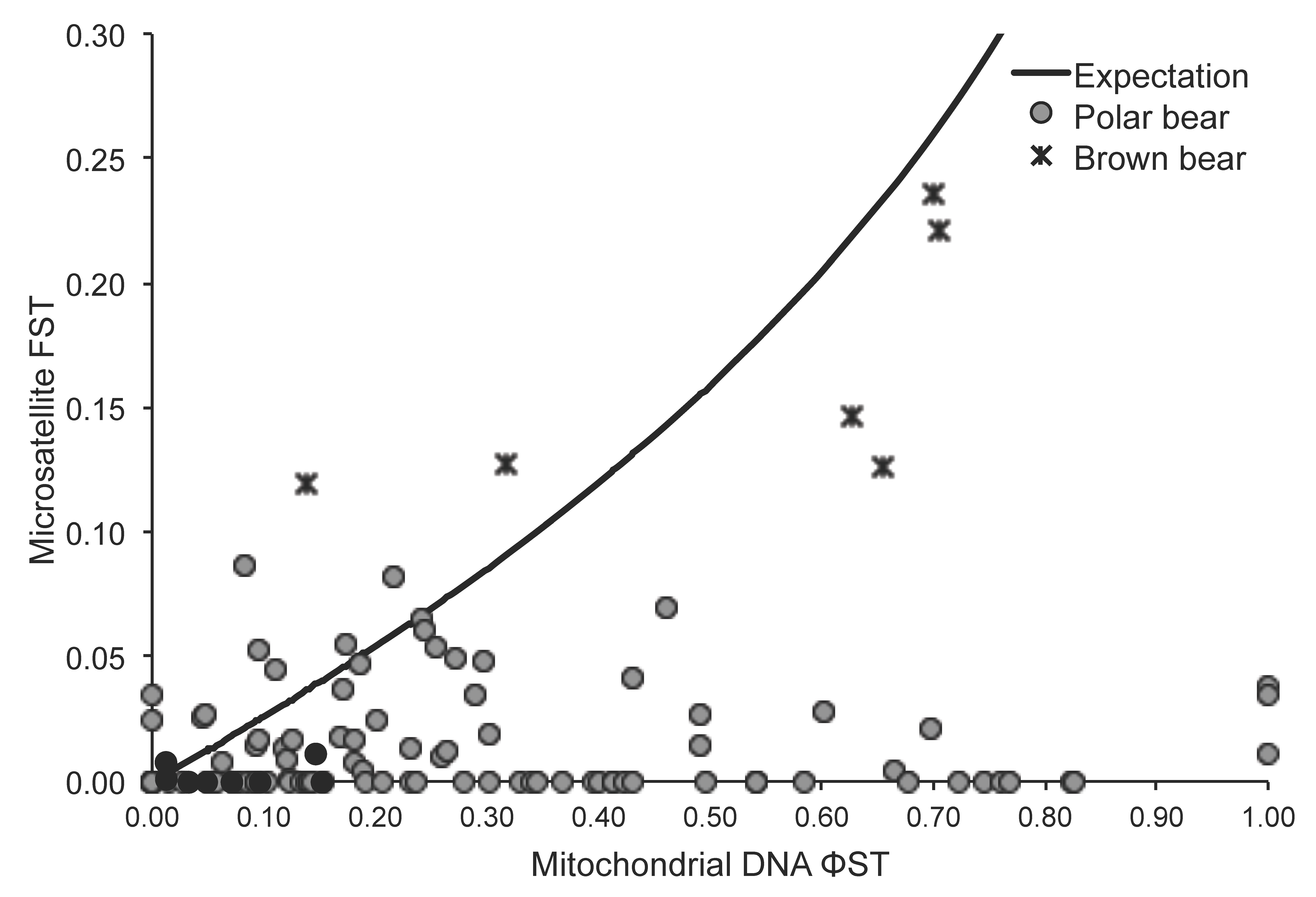

Supplement: S4 Fig — Scatter plot of observed pairwise mtDNA ΦST versus pairwise microsatellite F ST values for 21 microsatellite loci (circles) for 15 subpopulations of polar bears. The line represents the expected microsatellite F ST value given the genetic differentiation observed at mtDNA (2): F ST(nu) = 1–e 0.25*ln[1–FST(mt)]. Generally the pairwise comparisons are below the expectation line (i.e., lower FST derived from microsatellite markers compared with the mtDNA), which suggests higher female philopatry relative to males (i.e., male biased gene flow). Stars show similar comparisons of pairwise mtDNA ΦST and microsatellite F ST values for brown bear populations in Alaska. Black circles show values that represent polar bear subpopulations that are ≤900 kilometers between each other for comparison to the brown bear populations shown, which are 900 km apart from each other. (TIFF) [file pone.0112021.s004.tiff]

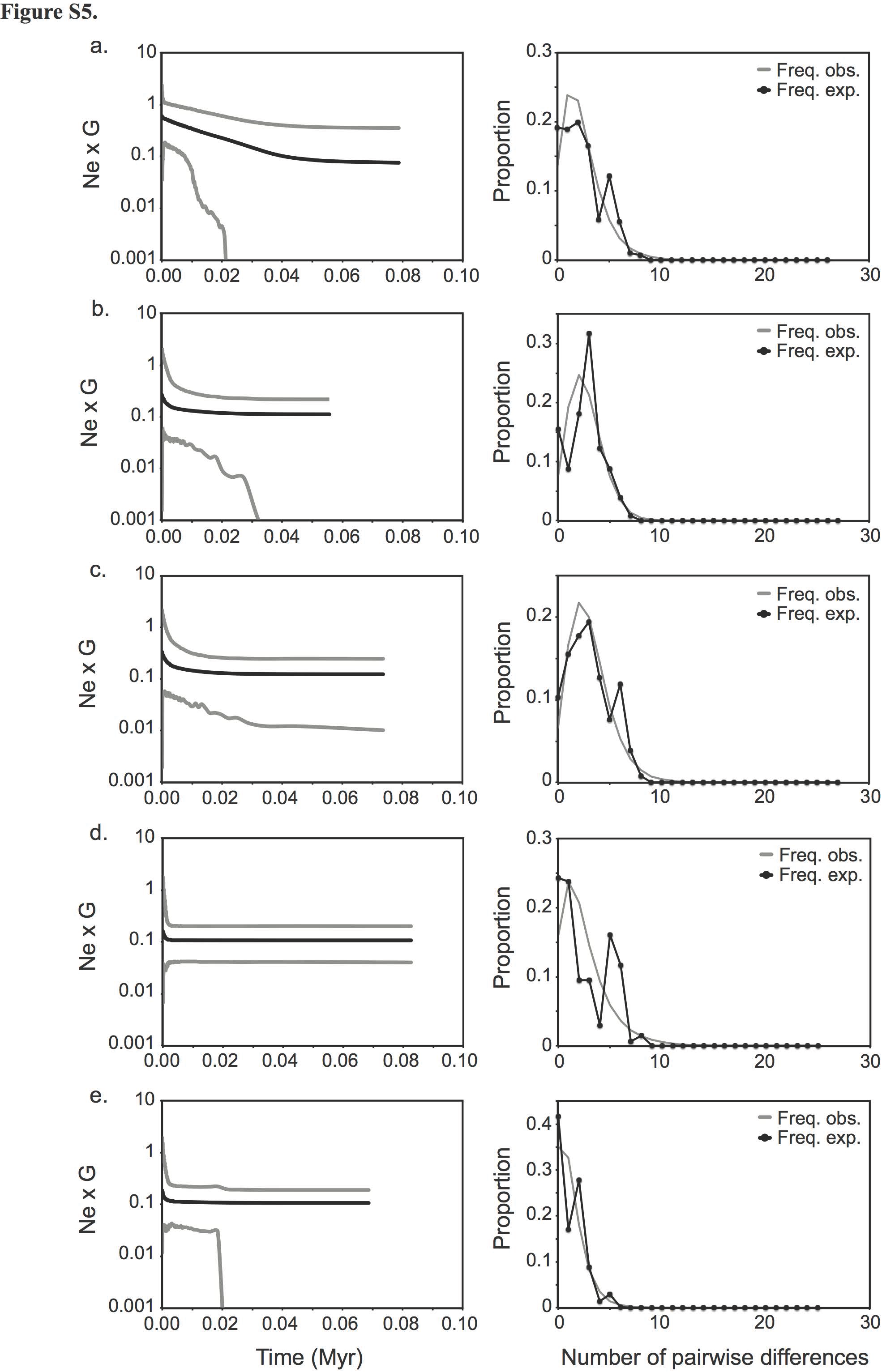

Supplement: S5 Fig — Extended Bayesian skyline plots (EBSPs) and pairwise mismatch distributions for (a) global-wide (b) Eastern Polar Basin, (c) Western Polar Basin, (d) Canadian Archipelago, and (e) Southern Canada clusters of polar bears. EBSPs indicate population growth from past (right) to present (left) including median population size through time (black line) and 95% highest probability distribution (grey interval). Log-transformed y-axes represent population size as a function of effective size (N e) and generation time (G). Mismatch distributions indicate the frequency of expected (grey line) and observed (black) pairwise differences. (TIFF) [file pone.0112021.s005.tiff]

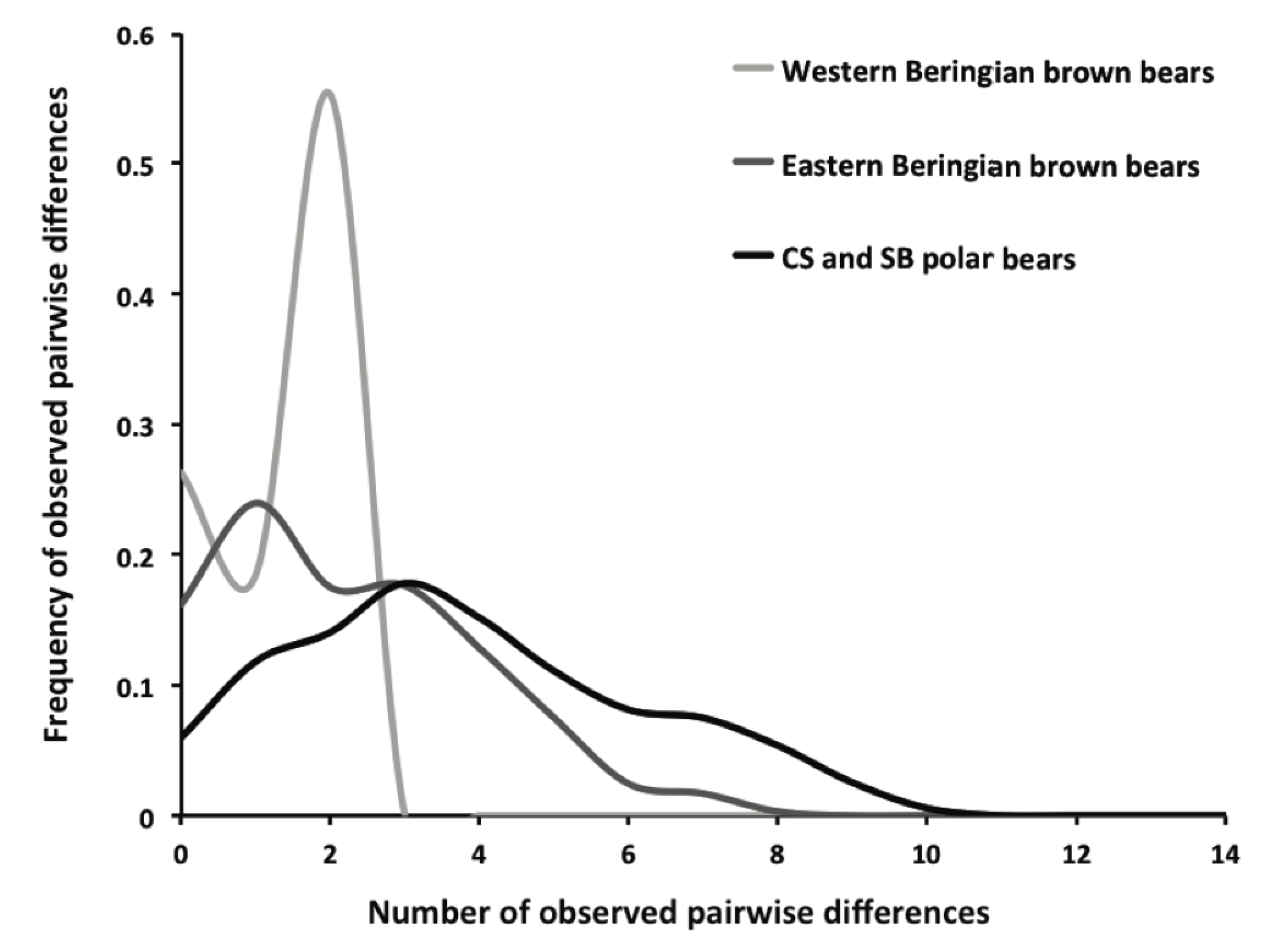

Supplement: S6 Fig — Mismatch distributions of pairwise differences based on mtDNA data of polar bears from the southern Beaufort (SB) and Chukchi Sea (CS) subpopulations and brown bears from the Western and Eastern Beringian Clades. Mismatch signals between the lineages are offset, signifying different periods of demographic growth. (TIF) [file pone.0112021.s006.tif]
