# Supplementary material for: Implications of the Circumpolar Genetic Structure of Polar Bears for Their Conservation in a Rapidly Warming Arctic
Source: PLoS One. 2015 Jan 6;10(1):e112021. doi: 10.1371/journal.pone.0112021 (PMC4285400; doi:10.1371/journal.pone.0112021)
Supplement: S1 Table — Estimates of genetic diversity in 18 circumpolar subpopulation of polar bears, arranged within the four genetic clusters identified in this paper, including allelic richness (AR), observed heterozygosity (Ho), expected heterozygosity (He) for the microsatellite data; and the number of haplotypes ( k ), haplotype ( h ) and nucleotide diversity ( π ), and Ewen-Wattersen's neutrality (E ν ) for the mitochondrial DNA control region. Genetic demographic statistics include the growth statistic (g) with standard deviation (bold if significant where g≥SD[g]), Fu's Fs, Tajima's D, raggedness (rg), and Deviation from a sudden expansion (SSD). Bold values for microsatellite data signify microsatellite data from subpopulations that were out of Hardy-Weinberg Equilibrium at α = 0.05/number of loci. Bold values for demographic statistics indicate significance at P≤0.05. Abbreviations of subpopulations are as follows: Baffin Bay (BB); Barents Sea (BS); Chukchi Sea (CS); Davis Strait (DS); East Greenland (EG); Foxe Basin (FB); Gulf of Boothia (GB); Kane Basin (KB); Kara Sea (KS); Laptev Sea (LP); Lancaster Sound (LS); M'Clintock Channel (MC); Northern Beaufort Sea (NB); Norwegian Bay (NW); Southern Beaufort Sea (SB); Southern Hudson Bay (SH); Viscount Melville (VM); and Western Hudson Bay (WH). (DOCX) [file pone.0112021.s007.docx]

**Table S1.** Estimates of genetic diversity in 18 circumpolar subpopulation of polar bears, arranged within the four genetic clusters identified in this paper, including allelic richness (AR), observed heterozygosity (H_o_), expected heterozygosity (H_e_) for the microsatellite data; and the number of haplotypes (*k*), haplotype (*h*) and nucleotide diversity (*π*), and Ewen-Wattersen’s neutrality (E*ν)* for the mitochondrial DNA control region. Genetic demographic statistics include the growth statistic (*g*) with standard deviation (bold if significant where *g* ≥ SD[*g*]), Fu’s *Fs*, Tajima’s *D*, raggedness (*rg*), and Deviation from a sudden expansion (SSD). Bold values for microsatellite data signify microsatellite data from subpopulations that were out of Hardy-Weinberg Equilibrium at α = 0.05/number of loci. Bold values for demographic statistics indicate significance at *P* ≤ 0.05. Abbreviations of subpopulations are as follows: Baffin Bay (BB); Barents Sea (BS); Chukchi Sea (CS); Davis Strait (DS); East Greenland (EG); Foxe Basin (FB); Gulf of Boothia (GB); Kane Basin (KB); Kara Sea (KS); Laptev Sea (LP); Lancaster Sound (LS); M’Clintock Channel (MC); Northern Beaufort Sea (NB); Norwegian Bay (NW); Southern Beaufort Sea (SB); Southern Hudson Bay (SH); Viscount Melville (VM); and Western Hudson Bay (WH).

| Subpopulation | Nuclear microsatellite | | | | | Mitochondrial DNA | | | | | | | | |  | Demographic Statistics | | |  |
| --- | --- | --- | --- | --- | --- | --- | --- | --- | --- | --- | --- | --- | --- | --- | --- | --- | --- | --- | --- |
|  | *n* | # of loci | | AR | H_o_/H_e_ | *n* | *k* | | | *h* | | *π* | | *Eν* | *g* (sd[*g*]) | *Fs* | *D* | *rg* | SSD |
| All Polar Bears | | |  | |  | 411 | | 50 | 0.889 | | 0.0051 | | - | | **915.33 (48.01)** | **-35.587** | -1.041 | **0.013** | 0.044 |
| Eastern Polar Basin Cluster | | |  | |  | 61 | | 15 | 0.845 | | 0.0046 | | - | | **341.80 (121.12)** | **-4.252** | -0.534 | - | - |
| EG | 30 | 16 | 5.50 | | 0.67/0.69 | - | | - | - | | - | | - | | - | - | - | - | - |
| BS | 455 | 15 | 5.53 | | 0.68/0.68 | 30 | | 9 | 0.892 | | 0.0054 | | 0.129 | | 416.95 (191.28) | -2.121 | 0.097 | 0.061 | 0.075 |
| KS | 17 | 21 | 5.06 | | 0.66/0.68 | 17 | | 7 | 0.838 | | 0.0044 | | 0.211 | | 490.60 (245.56) | -0.859 | -0.140 | 0.056 | 0.061 |
| LP | 15 | 21 | 5.72 | | 0.64/0.73 | 14 | | 10 | 0.945 | | 0.0060 | | 0.091 | | **756.92 (148.16)** | -3.966 | 0.379 | 0.048 | 0.055 |
| Western Polar Basin Cluster | | |  | |  | 65 | | 16 | 0.897 | | 0.0053 | | - | | **566.59 (159.09)** | -3.935 | -0.295 | **-** | - |
| CS | 266 | 21 | 5.61 | | 0.70/0.71 | 35 | | 16 | 0.918 | | 0.0060 | | 0.097 | | **723.11 (120.45)** | **-5.840** | -0.511 | **0.010** | 0.043 |
| SB | 233 | 20 | 5.67 | | 0.70/0.70 | 30 | | 15 | 0.936 | | 0.0070 | | 0.096 | | **447.07 (131.57)** | **-4.609** | 0.237 | 0.023 | 0.054 |
| NB | 30 | 16 | 5.68 | | 0.70/0.70 | - | | - | - | | - | | - | | - | - | - | - | - |
| Canadian Archipelago Cluster^*^ | | | | |  | 147 | | 13 | 0.756 | | 0.0043 | | - | | 239.85 (105.86) | -1.068 | -0.018 | - | - |
| VM | 30 | 16 | 5.53 | | 0.65/0.66 | 3 | | 1 | 0.000 | | 0.0000 | | - | | - | - | - | - | - |
| MC | 14 | 16 | 5.42 | | 0.70/0.68 | 2 | | 1 | 0.000 | | 0.0000 | | - | | - | - | - | - | - |
| GB | 47 | 21 | 5.94 | | 0.73/0.74 | 16 | | 5 | 0.608 | | 0.0030 | | 0.359 | | 27.32 (163.14) | -0.039 | -1.001 | 0.095 | 0.053 |
| LS | 65 | 21 | 5.74 | | 0.73/0.73 | 34 | | 10 | 0.845 | | 0.0066 | | 0.169 | | 237.55 (105.33) | -0.265 | 0.930 | **0.022** | 0.067 |
| NW | 31 | 16 | 5.40 | | 0.66/0.67 | 3 | | 1 | 0.000 | | 0.0000 | | - | | - | - | - | - | - |
| KB | 30 | 16 | 5.64 | | 0.71/0.71 | - | | - | - | | - | | - | | - | - | - | - | - |
| BB | 175 | 20 | 5.75 | | **0.73/0.73** | 30 | | 9 | 0.837 | | 0.0050 | | 0.147 | | 160.24 (150.18) | -0.748 | 0.528 | 0.039 | 0.063 |
| Southern Canada Cluster^*^ | | | | |  | 139 | | 13 | 0.538 | | 0.0021 | | - | | **939.36 (191.98)** | **-5.416** | **-1.474** | **-** | - |
| DS^*^ | 1050 | 20 | 5.61 | | 0.70/0.71 | 121 | | 15 | 0.769 | | 0.0033 | | 0.137 | | 168.38 (138.76) | **-4.493** | -0.568 | **0.024** | 0.057 |
| FB | 142 | 20 | 5.51 | | 0.69/0.70 | 27 | | 5 | 0.621 | | 0.0023 | | 0.314 | | 376.30 (439.22) | -0.026 | 0.041 | 0.077 | 0.067 |
| Subpopulation | Nuclear microsatellite | | | | | Mitochondrial DNA | | | | | | | | |  | Demographic Statistics | | |  |
|  | *n* | # of loci | | AR | H_o_/H_e_ | *n* | *k* | | | *h* | | *π* | | *Eν* | *g* (sd[*g*]) | *Fs* | *D* | *rg* | SSD |
| SH | 60 | 21 | 5.13 | | 0.69/0.69 | 23 | | 8 | 0.581 | | 0.0019 | | 0.444 | | **5213.9 (187.03)** | **-4.263** | **-1.842** | 0.078 | 0.032 |
| WH | 59 | 21 | 5.15 | | 0.68/0.70 | 26 | | 9 | 0.865 | | 0.0046 | | 0.152 | | 484.44 (227.30) | -1.485 | 0.375 | 0.032 | 0.052 |

**^*^**Clustering analysis presented in this paper splits the Davis Strait (DS) subpopulation, north and south of Hudson Strait, between the Southern Canada Cluster and the Canadian Archipelago Cluster. In this table, we arbitrarily present the genetic diversity statistics for DS within the Southern Canada Cluster subheading.
