# Supplementary material for: Implications of the Circumpolar Genetic Structure of Polar Bears for Their Conservation in a Rapidly Warming Arctic
Source: PLoS One. 2015 Jan 6;10(1):e112021. doi: 10.1371/journal.pone.0112021 (PMC4285400; doi:10.1371/journal.pone.0112021)
Supplement: S2 Table — Distribution of haplotypes (and GenBank accession numbers) at the mitochondrial DNA control region in 18 subpopulations of polar bears: Baffin Bay (BB); Barents Sea (BS); Chukchi Sea (CS); Davis Strait (DS); East Greenland (EG); Foxe Basin (FB); Gulf of Boothia (GB); Kane Basin (KB); Kara Sea (KS); Laptev Sea (LP); Lancaster Sound (LS); M'Clintock Channel (MC); Northern Beaufort Sea (NB); Norwegian Bay (NW); Southern Beaufort Sea (SB); Southern Hudson Bay (SH); Viscount Melville (VM); Western Hudson Bay (WH). (DOCX) [file pone.0112021.s008.docx]

**Table S2**. Distribution of haplotypes (and GenBank accession numbers) at the mitochondrial DNA control region in 18 subpopulations of polar bears: Baffin Bay (BB); Barents Sea (BS); Chukchi Sea (CS); Davis Strait (DS); East Greenland (EG); Foxe Basin (FB); Gulf of Boothia (GB); Kane Basin (KB); Kara Sea (KS); Laptev Sea (LP); Lancaster Sound (LS); M’Clintock Channel (MC); Northern Beaufort Sea (NB); Norwegian Bay (NW); Southern Beaufort Sea (SB); Southern Hudson Bay (SH); Viscount Melville (VM); Western Hudson Bay (WH).

| Subpopulation |  | BB | DS | FB | GB | LS | MC | NW | VM | WH | SH | CS | SB | KS | LP | BS |
| --- | --- | --- | --- | --- | --- | --- | --- | --- | --- | --- | --- | --- | --- | --- | --- | --- |
| Haplotype | GenBank Number |  |  |  |  |  |  |  |  |  |  |  |  |  |  |  |
|  |  |  |  |  |  |  |  |  |  |  |  |  |  |  |  |  |
| UMACR01 | KM676498 | 6 | 32 | 13 | 9 | 5 | – | 3 | – | 4 | 15 | 4 | 1 | – | 1 | 2 |
| UMACR02 | KM676499 | – | 1 | – | – | – | – | – | – | – | – | 2 | – | – | – | – |
| UMACR03 | KM676500 | – | 15 | – | – | – | – | – | – | 6 | 1 | – | – | – | 2 | 4 |
| UMACR05 | KM676501 | 2 | 3 | – | 2 | 11 | 2 | – | – | – | 1 | 1 | 2 | 1 | 2 | – |
| UMACR06 | KM676502 | – | – | – | – | – | – | – | – | – | 2 | 7 | – | 1 | – | – |
| UMACR07 | KM676503 | – | – | – | – | – | – | – | – | – | – | – | 1 | – | – | – |
| UMACR08 | KM676504 | – | – | – | – | – | – | – | – | – | – | 1 | 2 | – | – | 1 |
| UMACR09 | KM676505 | – | – | – | – | – | – | – | – | – | – | 2 | 2 | – | – | – |
| UMACR10 | KM676506 | – | – | – | – | – | – | – | – | – | 1 | 4 | – | – | – | – |
| UMACR11 | KM676507 | – | – | – | – | – | – | – | – | – | – | 1 | 3 | – | – | – |
| UMACR12 | KM676508 | 1 | – | – | – | 2 | – | – | – | – | – | – | 3 | – | 1 | – |
| UMACR14 | KM676509 | – | – | – | – | – | – | – | – | – | – | 1 | – | – | – | – |
| UMACR15 | KM676510 | – | – | – | – | – | – | – | – | – | – | – | – | 2 | 1 | – |
| UMACR16 | KM676511 | – | – | – | – | – | – | – | – | – | – | – | 1 | – | – | 2 |
| UMACR17 | KM676512 | – | – | – | – | – | – | – | – | – | – | 2 | 6 | 3 | 1 | 1 |
| UMACR18 | KM676513 | – | – | – | – | – | – | – | – | – | – | – | – | – | – | 3 |
| UMACR19 | KM676514 | – | – | – | – | 2 | – | – | – | – | – | 1 | 2 | 6 | – | 8 |
| UMACR20 | KM676515 | – | – | – | – | – | – | – | – | – | – | – | – | – | – | 2 |
| UMACR21 | KM676516 | 1 | 4 | – | – | 1 | – | – | – | – | – | – | – | 3 | – | 1 |
| Subpopulation |  | BB | DS | FB | GB | LS | MC | NW | VM | WH | SH | CS | SB | KS | LP | BS |
| Haplotype | GenBank Number |  |  |  |  |  |  |  |  |  |  |  |  |  |  |  |
| UMACR22 | KM676517 | – | – | – | – | – | – | – | – | – | – | – | – | – | – | 1 |
| UMACR23 | KM676518 | – | – | – | – | – | – | – | – | – | – | – | – | – | – | 3 |
| UMACR25 | KM676519 | – | 1 | – | – | – | – | – | – | – | – | – | – | – | 1 | – |
| UMACR26 | KM676520 | – | – | – | – | – | – | – | – | – | – | – | – | – | 1 | 1 |
| UMACR28 | KM676521 | 1 | 4 | 1 | 2 | – | – | – | – | 1 | – | – | 1 | – | 1 | – |
| UMACR29 | KM676522 | – | – | – | – | – | – | – | – | – | – | – | – | – | – | 1 |
| UMACR32 | KM676523 | – | 1 | 1 | – | – | – | – | – | 5 | 1 | – | – | – | – | – |
| UMACR34 | KM676524 | – | 1 | – | – | – | – | – | – | – | – | – | – | – | – | – |
| UMACR35 | KM676525 | – | 4 | – | – | – | – | – | – | – | – | – | – | – | – | – |
| UMACR36 | KM676526 | – | 21 | 3 | 1 | – | – | – | – | – | – | – | – | – | – | – |
| UMACR37 | KM676527 | – | 5 | – | – | – | – | – | – | – | – | – | – | – | – | – |
| UMACR38 | KM676528 | 1 | 13 | – | – | – | – | – | – | – | – | – | – | – | – | – |
| UMACR39 | KM676529 | 5 | 1 | 4 | 1 | 5 | – | – | – | – | – | – | – | – | – | – |
| UMACR40 | KM676530 | 3 | 1 | – | – | – | – | – | – | – | – | – | – | – | – | – |
| UMACR41 | KM676531 | 7 | – | – | – | 3 | – | – | – | 2 | – | – | – | – | – | – |
| UMACR42 | KM676532 | 2 | – | – | – | 2 | – | – | – | – | – | – | – | – | – | – |
| UMACR43 | KM676533 | – | 8 | 4 | – | – | – | – | – | 1 | – | – | – | – | – | – |
| UMACR44 | KM676534 | 1 | 1 | – | – | – | – | – | – | – | – | – | – | – | – | – |
| UMACR45 | KM676535 | – | – | – | – | – | – | – | 3 | – | – | – | – | – | – | – |
| UMACR46 | KM676536 | – | – | – | 1 | – | – | – | – | – | – | – | – | – | – | – |
| UMACR48 | KM676537 | – | – | – | – | – | – | – | – | – | – | – | 3 | – | – | – |
| UMACR57 | KM676538 | – | 2 | – | – | – | – | – | – | 1 | – | – | 1 | – | – | – |
| UMACR58 | KM676539 | – | – | – | – | – | – | – | – | – | – | – | – | – | 1 | – |
| UMACR59 | KM676540 | – | – | – | – | – | – | – | – | – | – | 4 | – | – | – | – |
| UMACR60 | KM676541 | – | – | – | – | – | – | – | – | – | – | 1 | – | – | – | – |
| Subpopulation |  | BB | DS | FB | GB | LS | MC | NW | VM | WH | SH | CS | SB | KS | LP | BS |
| Haplotype | GenBank Number |  |  |  |  |  |  |  |  |  |  |  |  |  |  |  |
| UMACR61 | KM676542 | – | – | – | – | – | – | – | – | – | – | – | 1 | – | – | – |
| UMACR63 | KM676543 | – | – | – | – | – | – | – | – | – | – | – | 1 | – | – | – |
| UMACR66 | KM676544 | – | – | – | – | 1 | – | – | – | – | – | – | – | – | – | – |
| UMACR67 | KM676545 | – | – | – | – | 1 | – | – | – | – | – | – | – | – | – | – |
| UMACR68 | KM676546 | – | – | – | – | 1 | – | – | – | – | – | – | – | – | – | – |
| UMACR69 | KM676547 | – | – | – | – | – | – | – | – | 5 | – | – | – | – | – | – |
| UMACR70 | KM676548 | – | – | – | – | – | – | – | – | 1 | – | – | – | – | – | – |
| UMACR71 | KM676549 | – | 1 | – | – | – | – | – | – | – | – | – | – | – | – | – |
| UMACR73 | KM821364 | – | 1 | – | – | – | – | – | – | – | – | – | – | – | – | – |
| UMACR74 | KM676550 | – | 1 | – | – | – | – | – | – | – | – | – | – | – | – | – |
| UMACR75 | KM676551 | – | – | – | – | – | – | – | – | – | – | – | – | 1 | – | – |
| UMACR81 | KM676552 | – | – | – | – | – | – | – | – | – | – | 1 | – | – | – | – |
| UMACR82 | KM676553 | – | – | – | – | – | – | – | – | – | 1 | – | – | – | – | – |
| UMACR83 | KM676554 | – | – | – | – | – | – | – | – | – | 1 | – | – | – | – | – |
| UMACR84 | KM676555 | – | – | – | – | – | – | – | – | – | – | 1 | – | – | – | – |
| UMACR85 | KM676556 | – | – | – | – | – | – | – | – | – | – | 1 | – | – | 1 | – |
| UMACR86 | KM676557 | – | – | – | – | – | – | – | – | – | – | 1 | – | – | – | – |
| UMACR87 | KM676558 | – | – | – | – | – | – | – | – | – | – | – | – | – | 1 | – |
| UMACR88 | KM676559 | – | – | – | – | – | – | – | – | 1 | – | – | – | – | – | – |
|  |  |  |  |  |  |  |  |  |  |  |  |  |  |  |  |  |
| Total (N) |  | 30 | 121 | 27 | 16 | 34 | 2 | 3 | 3 | 26 | 23 | 35 | 30 | 17 | 14 | 30 |
| *k* |  | 11 |  | 6 | 6 | 11 | 1 | 1 | 1 | 10 | 8 | 17 | 15 | 7 | 12 | 13 |
|  |  |  |  |  |  |  |  |  |  |  |  |  |  |  |  |  |
