# Supplementary material for: Implications of the Circumpolar Genetic Structure of Polar Bears for Their Conservation in a Rapidly Warming Arctic
Source: PLoS One. 2015 Jan 6;10(1):e112021. doi: 10.1371/journal.pone.0112021 (PMC4285400; doi:10.1371/journal.pone.0112021)
Supplement: S3 Table — Genetic differentiation results of comparisons of microsatellite data from earlier and later samples within nine global subpopulations and regions of polar bears: the Svalbard portion of the Barents Sea (BS); Baffin Bay (BB); Chukchi Sea (CS); Foxe Basin (FB); Gulf of Boothia (GB); the Labrador portion of Davis Strait (DS); Lancaster Sound (LS); Southern Beaufort Sea (SB) and Western Hudson Bay (WH). Degrees of freedom are shown in parentheses. The K metric represents the likely number of clusters for the subpopulation or region with decadal data pooled as ascertained using the Bayesian clustering program baps. Values in bold show significant differentiation between the groups (α = 0.05, Bonferroni corrections applied). (DOCX) [file pone.0112021.s009.docx]

**Table S3.** Genetic differentiation results of comparisons of microsatellite data from earlier and later samples within nine global subpopulations and regions of polar bears: the Svalbard portion of the Barents Sea (BS); Baffin Bay (BB); Chukchi Sea (CS); Foxe Basin (FB); Gulf of Boothia (GB); the Labrador portion of Davis Strait (DS); Lancaster Sound (LS); Southern Beaufort Sea (SB) and Western Hudson Bay (WH). Degrees of freedom are shown in parentheses. The K metric represents the likely number of clusters for the subpopulation or region with decadal data pooled as ascertained using the Bayesian clustering program baps. Values in bold show significant differentiation between the groups (α = 0.05, Bonferroni corrections applied).

| Decadal comparison | Genic differentiation χ^2^ | Genotypic differentiation χ^2^ | baps |
| --- | --- | --- | --- |
| BB 1995 vs. 2007 | 38.65 (32) | 37.84 (32) | K = 1, Ln = -3157.47, LnP = 1.0 |
| Svalbard 1992-96 vs. 2004-06 | 28.79 (30) | 27.99 (30) | K = 1, Ln = -2874.80, LnP = 1.0 |
| CS 1986-90 vs. 2011 | 61.77 (42) | 51.84 (42) | K = 1, Ln = -3984.08, LnP = 1.0 |
| FB 1991-97 vs. 2008 | 42.20 (32) | 41.68 (32) | K = 1, Ln = -2986.04, LnP = 1.0 |
| GB 1992-96 vs. 2007-08 | 34.87 (30) | 33.58 (30) | K = 1, Ln = -2807.86, LnP = 1.0 |
| Labrador 1991-94 vs. 2007 | 38.47 (32) | 38.34 (32) | K = 1, Ln = -2691.27, LnP = 1.0 |
| LS 1985-95 vs. 2008-10 | 43.10 (32) | 45.51 (32) | K = 1, Ln = -3182.54, LnP = 1.0 |
| SB 1982-87 vs. 2010 | 26.61 (40) | 30.18 (40) | K =1, Ln = -3999.93, LnP = 1.0 |
| WH 1986-89 vs. 2008 | 32.51 (32) | 31.40 (32) | K = 1, Ln = -2168.05, LnP = 1.0 |
