# Supplementary material for: Implications of the Circumpolar Genetic Structure of Polar Bears for Their Conservation in a Rapidly Warming Arctic
Source: PLoS One. 2015 Jan 6;10(1):e112021. doi: 10.1371/journal.pone.0112021 (PMC4285400; doi:10.1371/journal.pone.0112021)
Supplement: S4 Table — Below the diagonal, significant (+) and non-significant (−) differences for genic differentiation, FST, and genotypic differentiation (the latter only for comparisons of subpopulations that were found to be out of Hardy-Weinberg equilibrium) for pairwise comparisons of 18 subpopulations of polar bears using microsatellite data. Bonferroni-corrected significance levels were adjusted for the number of loci compared for each pair (0.05/number of loci compared). Shaded blocks delineate the four clusters based on our analysis: Eastern Polar Basin, Western Polar Basin, Canadian Archipelago and Southern Canada. Above the diagonal: significant (+) and non-significant (−) differences for FST, θST and haplotypic differentiation using mtDNA data for pairwise comparisons of 15 subpopulations. Abbreviations for subpopulations are as follows: Baffin Bay (BB); Barents Sea (BS); Chukchi Sea (CS); Davis Strait (DS); East Greenland (EG); Foxe Basin (FB); Gulf of Boothia (GB); Kane Basin (KB); Kara Sea (KS); Laptev Sea (LP); Lancaster Sound (LS); M'Clintock Channel (MC); Northern Beaufort Sea (NB); Norwegian Bay (NW); Southern Beaufort Sea (SB); Southern Hudson Bay (SH); Viscount Melville (VM); and Western Hudson Bay (WH). (DOCX) [file pone.0112021.s010.docx]

**Table S4.** Below the diagonal, significant (+) and non-significant (-) differences for genic differentiation, F_ST_, and genotypic differentiation (the latter only for comparisons of subpopulations that were found to be out of Hardy-Weinberg equilibrium) for pairwise comparisons of 18 subpopulations of polar bears using microsatellite data. Bonferroni-corrected significance levels were adjusted for the number of loci compared for each pair (0.05/number of loci compared). Shaded blocks delineate the four clusters based on our analysis: Eastern Polar Basin, Western Polar Basin, Canadian Archipelago and Southern Canada. Above the diagonal: significant (+) and non-significant (-) differences for F_ST_, Θ_ST_ and haplotypic differentiation using mtDNA data for pairwise comparisons of 15 subpopulations. Abbreviations for subpopulations are as follows: Baffin Bay (BB); Barents Sea (BS); Chukchi Sea (CS); Davis Strait (DS); East Greenland (EG); Foxe Basin (FB); Gulf of Boothia (GB); Kane Basin (KB); Kara Sea (KS); Laptev Sea (LP); Lancaster Sound (LS); M’Clintock Channel (MC); Northern Beaufort Sea (NB); Norwegian Bay (NW); Southern Beaufort Sea (SB); Southern Hudson Bay (SH); Viscount Melville (VM); and Western Hudson Bay (WH).

|  | EG | BS | KS | LP | CS | SB | NB | VM | MC | GB | LS | NW | KB | BB | DS | FB | SH | WH |
| --- | --- | --- | --- | --- | --- | --- | --- | --- | --- | --- | --- | --- | --- | --- | --- | --- | --- | --- |
| EG |  |  |  |  |  |  |  |  |  |  |  |  |  |  |  |  |  |  |
| BS | -,- |  | +,+,+ | +,+,+ | +,+,+ | +,+,+ |  | +,+,+ | +,+,+ | +,+,+ | +,+,+ | +,-,+ |  | +,+,+ | +,+,+ | +,+,+ | +,+,+ | +,+,+ |
| KS | -,- | -,- |  | +,+,+ | +,+,+ | +,+,+ |  | +,+,+ | +,+,+ | +,+,+ | +,+,+ | +,+,+ |  | +,+,+ | +,+,+ | +,+,+ | +,+,+ | +,+,+ |
| LP | +,-, | -,- | +,- |  | +,-,+ | +,-,+ |  | +,+,+ | -,-,- | +,+,+ | -,-,+ | +,-,+ |  | +,+,+ | +,+,+ | +,+,+ | +,+,+ | +,+,+ |
| CS | +, - | +, - | +, + | +,- |  | +,-,+ |  | +,+,+ | +,+,+ | +,+,+ | +,+,+ | +,-,+ |  | +,+,+ | +,+,+ | +,+,+ | +,+,+ | +,+,+ |
| SB | +,- | +, - | +,- | -,- | +, -, + |  |  | +,+,+ | +,-,+ | +,+,+ | +,-,+ | +,-,+ |  | +,+,+ | +,+,+ | +,+,+ | +,+,+ | +,+,+ |
| NB | +, +, | +, - | +,- | +,- | +,- | +,- |  |  |  |  |  |  |  |  |  |  |  |  |
| VM | +, + | +, - | +,- | +,- | +, - | +,- | +,+ |  | -,-,+ | +,+,+ | +,+,+ | -,-,+ |  | +,+,+ | +,+,+ | +,+,+ | +,+,+ | +,+,+ |
| MC | +,+ | +, - | +,- | +,- | +,- | +,+ | +,+ | -,- |  | +,+,+ | -,-,- | -,-,+ |  | +,-,+ | +,+,+ | +,+,+ | +,+,+ | +,+,+ |
| GB | +, + | +, - | +, - | +,+ | +, - | +, + | +, + | +, - | +,- |  | +,+,+ | -,-,- |  | +,-,+ | +,+,+ | -,-,+ | -,-,+ | +,+,+ |
| LS | +,- | +, - | +, + | +,+ | +, + | +, + | +,- | +,- | -,- | +, + |  | +,-,+ |  | +,-,+ | +,+,+ | +,+,+ | +,+,+ | +,+,+ |
| NW | +, + | +, - | +,- | +,- | +, -, + | +,- | +, + | +, + | +,+ | +, + | +,- |  |  | +,-,+ | -,-,- | -,-,- | -,-,- | +,-,+ |
| KB | +, + | +, - | +,- | +,- | +, - | +,- | +,+ | +,+ | +,- | +, - | +,- | +,+ |  |  |  |  |  |  |
| BB | +, - | +, - | +, + | +,+ | +, +,+ | +, +, + | +,- | +, - | +,- | +, - | +,- | +, -, + | -,- |  | +,+,+ | +,+,+ | +,+,+ | +,+,+ |
| DS | +, - | +, - | +,+ | +,+ | +, + | +, + | +, - | +, - | +, - | +, - | +, - | +, - | +, - | +,+ |  | +,+,+ | +,+,+ | +,+,+ |
| FB | +, - | +, - | +, + | +, + | +, + | +, + | +,- | +,- | +,- | +, + | +, + | +,- | +, - | +,+ | +, - |  | -,-,+ | +,+,+ |
| SH | +,- | +, - | +, + | +,+ | +, + | +, + | +,- | +,- | +,+ | +, - | +,+ | +,- | +,- | +,- | +, - | +,- |  | +,+,+ |
| WH | +, + | +, - | +, + | +, + | +, + | +, + | +,+ | +,+ | +, + | +, + | +, + | +, + | +, + | +,+ | +, - | +,- | +,- |  |
