# Supplementary material for: Implications of the Circumpolar Genetic Structure of Polar Bears for Their Conservation in a Rapidly Warming Arctic
Source: PLoS One. 2015 Jan 6;10(1):e112021. doi: 10.1371/journal.pone.0112021 (PMC4285400; doi:10.1371/journal.pone.0112021)
Supplement: S5 Table — Pairwise estimates of population differentiation among 15 (mitochondrial DNA) or 18 (microsatellite DNA) circumpolar subpopulations of polar bears: Baffin Bay (BB); Barents Sea (BS); Chukchi Sea (CS); Davis Strait (DS); East Greenland (EG); Foxe Basin (FB); Gulf of Boothia (GB); Kane Basin (KB); Kara Sea (KS); Laptev Sea (LP); Lancaster Sound (LS); M'Clintock Channel (MC); Northern Beaufort Sea (NB); Norwegian Bay (NW); Southern Beaufort Sea (SB); Southern Hudson Bay (SH); Viscount Melville (VM); Western Hudson Bay (WH). Significant values (α = 0.002 and 0.05 for microsatellite and mtDNA comparisons, respectively) are in bold text. (DOCX) [file pone.0112021.s011.docx]

**Table S5.** Pairwise estimates of population differentiation among 15 (mitochondrial DNA) or 18 (microsatellite DNA) circumpolar subpopulations of polar bears: Baffin Bay (BB); Barents Sea (BS); Chukchi Sea (CS); Davis Strait (DS); East Greenland (EG); Foxe Basin (FB); Gulf of Boothia (GB); Kane Basin (KB); Kara Sea (KS); Laptev Sea (LP); Lancaster Sound (LS); M’Clintock Channel (MC); Northern Beaufort Sea (NB); Norwegian Bay (NW); Southern Beaufort Sea (SB); Southern Hudson Bay (SH); Viscount Melville (VM); Western Hudson Bay (WH). Significant values (α = 0.002 and 0.05 for microsatellite and mtDNA comparisons, respectively) are in bold text.

| Subpopulation | Nuclear DNA | | | Mitochondrial DNA | | |
| --- | --- | --- | --- | --- | --- | --- |
|  | Fst | Rst | Genic differentiation (χ2) | Fst | Θst | χ2 |
| BB - |  |  |  |  |  |  |
| BS | 0.000 | -0.074 | **Infinity** | **0.147** | **0.078** | **Infinity** |
| CS | -0.113 | 0.001 | **Infinity*** | **0.115** | **0.080** | **Infinity** |
| DS | **0.018** | **0.007** | **Infinity** | **0.140** | **0.170** | **Infinity** |
| EG | **0.004** | -0.025 | **Infinity** | **-** | **-** | **-** |
| FB | -0.058 | **0.023** | **Infinity** | **0.110** | **0.106** | **Infinity** |
| GB | **0.017** | **0.071** | **Infinity** | **0.167** | 0.050 | **Infinity** |
| KB | -0.013 | -0.051 | 52.52 | **-** | **-** | **-** |
| KS | -0.086 | **0.024** | **Infinity** | **0.202** | **0.228** | **Infinity** |
| LP | **0.010** | 0.010 | **143.70** | **0.097** | **0.101** | **Infinity** |
| LS | **0.030** | -0.013 | **Infinity** | **0.050** | 0.044 | **Infinity** |
| MC | -0.010 | -0.064 | **114.26** | **0.296** | 0.501 | **10.90** |
| NB | -0.077 | -0.043 | **Infinity** | **-** | **-** | **-** |
| NW | -0.054 | **-**0.045 | **Infinity*** | **0.257** | 0.008 | **7.40** |
| SB | -0.060 | **0.027** | **Infinity*** | **0.141** | **0.100** | **Infinity** |
| SH | **0.045** | 0.006 | **Infinity** | **0.187** | **0.117** | **Infinity** |
| Subpopulation | Nuclear DNA | | | Mitochondrial DNA | | |
|  | Fst | Rst | Genic differentiation (χ2) | Fst | Θst | χ2 |
| BB - VM | 0.000 | -0.026 | **Infinity** | **0.399** | **0.450** | **Infinity** |
| WH | -0.063 | **0.033** | **Infinity** | **0.151** | **0.142** | **Infinity** |
| BS - |  |  |  |  |  |  |
| CS | -0.177 | -0.171 | **Infinity** | **0.068** | **0.061** | **Infinity** |
| DS | -0.113 | -0.076 | **Infinity** | **0.104** | **0.202** | **Infinity** |
| EG | -0.126 | -0.160 | 46.64 | **-** | **-** | **-** |
| FB | -0.080 | -0.049 | **Infinity** | **0.192** | **0.170** | **Infinity** |
| GB | -0.064 | **0.040** | **Infinity** | **0.235** | **0.118** | **Infinity** |
| KB | -0.073 | -0.092 | **Infinity** | **-** | **-** | **-** |
| KS | -0.263 | -0.229 | 43.67 | **0.055** | **0.062** | **Infinity** |
| LP | -0.190 | -0.179 | 53.94 | **0.061** | **0.069** | **Infinity** |
| LS | -0.081 | -0.068 | **Infinity** | **0.136** | **0.128** | **Infinity** |
| MC | -0.078 | -0.072 | **Infinity** | **0.282** | **0.502** | **Infinity** |
| NB | -0.111 | -0.139 | **Infinity** | **-** | **-** | **-** |
| NW | -0.042 | -0.019 | **Infinity** | **0.295** | 0.050 | **14.26** |
| SB | -0.135 | -0.129 | **Infinity** | **0.051** | **0.068** | **Infinity** |
| SH | -0.146 | -0.092 | **Infinity** | **0.219** | **0.147** | **Infinity** |
| VM | -0.077 | -0.028 | **Infinity** | **0.333** | **0.334** | **Infinity** |
| WH | -0.027 | 0.005 | **Infinity** | **0.101** | **0.177** | **Infinity** |
| CS - |  |  |  |  |  |  |
| DS | **0.027** | **0.013** | **Infinity** | **0.110** | **0.171** | **Infinity** |
| Subpopulation | Nuclear DNA | | | Mitochondrial DNA | | |
|  | Fst | Rst | Genic differentiation (χ2) | Fst | Θst | χ2 |
| CS - EG | -0.124 | -0.096 | **Infinity** | **-** | **-** | **Infinity** |
| FB | **0.041** | **0.031** | **Infinity** | **0.149** | **0.159** | **Infinity** |
| GB | -0.004 | **0.096** | **Infinity** | **0.180** | **0.064** | **Infinity** |
| KB | -0.087 | -0.074 | **Infinity** | **-** | **-** | **Infinity** |
| KS | **0.008** | **0.029** | **Infinity** | **0.112** | **0.159** | **Infinity** |
| LP | 0.003 | 0.007 | **85.48** | **0.040** | 0.005 | **Infinity** |
| LS | **0.020** | **0.014** | **Infinity** | **0.105** | **0.091** | **Infinity** |
| MC | -0.106 | -0.102 | **Infinity** | **0.220** | **0.399** | **11.14** |
| NB | -0.142 | -0.103 | **95.35** | **-** | **-** | **Infinity** |
| NW | -0.071 | -0.059 | **Infinity*** | **0.240** | 0.007 | **7.33** |
| SB | -0.025 | -0.029 | **Infinity*** | **0.044** | 0.035 | **Infinity** |
| SH | **0.087** | **0.062** | **Infinity** | **0.154** | **0.095** | **Infinity** |
| VM | -0.100 | -0.072 | **Infinity** | **0.308** | **0.429** | **Infinity** |
| WH | **0.051** | **0.061** | **Infinity** | **0.099** | **0.162** | **Infinity** |
| DS - |  |  |  |  |  |  |
| EG | -0.075 | -0.029 | **Infinity** | **-** | **-** | **-** |
| FB | 0.001 | 0.002 | **Infinity** | **0.099** | **0.078** | **Infinity** |
| GB | -0.022 | **0.066** | **Infinity** | **0.124** | **0.118** | **Infinity** |
| KB | -0.101 | -0.062 | **Infinity** | **-** | **-** | **-** |
| KS | **0.014** | 0.009 | **Infinity** | **0.199** | **0.423** | **Infinity** |
| LP | **0.038** | 0.010 | **Infinity** | **0.054** | **0.247** | **Infinity** |
| Subpopulation | Nuclear DNA | | | Mitochondrial DNA | | |
|  | Fst | Rst | Genic differentiation (χ2) | Fst | Θst | χ2 |
| DS - LS | -0.012 | -0.011 | **Infinity** | **0.158** | **0.314** | **Infinity** |
| MC | -0.098 | -0.062 | **150.08** | **0.325** | **0.708** | **Infinity** |
| NB | -0.072 | -0.065 | **Infinity** | **-** | **-** | **-** |
| NW | -0.081 | -0.037 | **Infinity** | **0.168** | 0.024 | 3.63 |
| SB | **0.049** | **0.030** | **Infinity** | **0.133** | **0.277** | **Infinity** |
| SH | -0.008 | -0.006 | **Infinity** | **0.101** | **0.116** | **Infinity** |
| VM | -0.083 | -0.039 | **Infinity** | **0.376** | **0.616** | **Infinity** |
| WH | -0.013 | 0.002 | **Infinity** | **0.044** | **0.091** | **Infinity** |
| EG - |  |  |  |  |  |  |
| FB | -0.023 | **0.019** | **Infinity** | **-** | **-** | **-** |
| GB | **0.020** | **0.088** | **Infinity** | **-** | **-** | **-** |
| KB | **0.030** | **0.039** | **Infinity** | **-** | **-** | **-** |
| KS | -0.161 | -0.085 | 51.30 | **-** | **-** | **-** |
| LP | -0.107 | -0.095 | **57.95** | **-** | **-** | **-** |
| LS | -0.014 | 0.009 | **Infinity** | **-** | **-** | **-** |
| MC | **0.029** | 0.014 | **98.67** | **-** | **-** | **-** |
| NB | **0.014** | 0.015 | **87.29** | **-** | **-** | **-** |
| NW | **0.055** | **0.053** | **Infinity** | **-** | **-** | **-** |
| SB | -0.064 | -0.039 | **Infinity** | **-** | **-** | **-** |
| SH | -0.081 | -0.038 | **Infinity** | **-** | **-** | **-** |
| VM | **0.038** | **0.056** | **Infinity** | **-** | **-** | **-** |
| Subpopulation | Nuclear DNA | | | Mitochondrial DNA | | |
|  | Fst | Rst | Genic differentiation (χ2) | Fst | Θst | χ2 |
| EG - WH | **0.052** | **0.088** | **Infinity** | **-** | **-** | **-** |
| FB - |  |  |  |  |  |  |
| GB | **0.008** | **0.096** | **Infinity** | 0.022 | 0.041 | **13.22** |
| KB | -0.054 | -0.026 | **Infinity** | **-** | **-** | **-** |
| KS | **0.027** | 0.017 | **Infinity** | **0.277** | **0.450** | **Infinity** |
| LP | **0.059** | 0.026 | **Infinity** | **0.147** | **0.265** | **Infinity** |
| LS | **0.012** | **0.009** | **Infinity** | **0.177** | **0.243** | **Infinity** |
| MC | -0.049 | -0.019 | **Infinity** | **0.445** | **0.805** | **Infinity** |
| NB | -0.020 | -0.034 | **Infinity** | **-** | **-** | - |
| NW | -0.036 | 0.003 | **Infinity** | 0.046 | 0.087 | 4.01 |
| SB | **0.066** | **0.052** | **Infinity** | **0.176** | **0.226** | **Infinity** |
| SH | -0.028 | -0.021 | **185.37** | 0.030 | 0.032 | **Infinity** |
| VM | -0.037 | -0.015 | **Infinity** | **0.481** | **0.766** | **Infinity** |
| WH | -0.010 | -0.002 | **138.47** | **0.112** | **0.058** | **Infinity** |
| GB - |  |  |  |  |  |  |
| KB | 0.003 | **0.055** | **Infinity** | **-** | **-** | **-** |
| KS | -0.005 | **0.130** | **Infinity** | **0.330** | **0.365** | **Infinity** |
| LP | **0.016** | **0.060** | **132.09** | **0.166** | **0.137** | **Infinity** |
| LS | **0.011** | **0.073** | **Infinity** | **0.194** | **0.136** | **Infinity** |
| MC | -0.007 | **0.053** | **Infinity** | **0.492** | **0.705** | **8.91** |
| NB | **0.015** | **0.088** | **Infinity** | **-** | **-** | **-** |
| Subpopulation | Nuclear DNA | | | Mitochondrial DNA | | |
|  | Fst | Rst | Genic differentiation (χ2) | Fst | Θst | χ2 |
| GB - NW | **0.024** | **0.080** | **Infinity** | -0.070 | 0.153 | 2.76 |
| SB | **0.013** | **0.099** | **Infinity** | **0.216** | **0.111** | **Infinity** |
| SH | -0.018 | **0.055** | **Infinity** | -0.021 | **-**0.027 | **15.60** |
| VM | 0.004 | **0.074** | **Infinity** | **0.583** | **0.712** | **Infinity** |
| WH | **0.053** | **0.117** | **Infinity** | **0.185** | **0.092** | **Infinity** |
| KB - |  |  |  |  |  |  |
| KS | -0.112 | -0.050 | **130.00** | **-** | **-** | **-** |
| LP | -0.055 | -0.065 | **Infinity** | **-** | **-** | **-** |
| LS | -0.039 | -0.035 | **70.46** | **-** | **-** | **-** |
| MC | 0.012 | -0.007 | **70.63** | **-** | **-** | **-** |
| NB | **0.038** | 0.014 | **Infinity** | **-** | **-** | **-** |
| NW | **0.023** | 0.008 | **Infinity** | **-** | **-** | **-** |
| SB | -0.034 | -0.024 | **Infinity** | **-** | **-** | **-** |
| SH | -0.119 | -0.071 | **Infinity** | **-** | **-** | **-** |
| VM | **0.025** | 0.022 | **104.21** | **-** | **-** | **-** |
| WH | **0.021** | **0.033** | **Infinity** | **-** | **-** | **-** |
| KS - |  |  |  |  |  |  |
| LP | 0.004 | **0.024** | **50.82** | **0.088** | **0.126** | **Infinity** |
| LS | **0.017** | 0.042 | **Infinity** | 0.168 | **0.167** | **Infinity** |
| MC | -0.130 | -0.064 | **90.93** | **0.324** | **0.536** | **12.12** |
| NB | -0.157 | -0.101 | **61.51** | **-** | **-** | **-** |
| Subpopulation | Nuclear DNA | | | Mitochondrial DNA | | |
|  | Fst | Rst | Genic differentiation (χ2) | Fst | Θst | χ2 |
| KS - NW | -0.086 | -0.004 | **Infinity** | **0.409** | **0.377** | **Infinity** |
| SB | -0.021 | -0.013 | **Infinity** | **0.129** | **0.123** | **Infinity** |
| SH | **0.070** | **0.067** | **Infinity** | **0.314** | **0.423** | **Infinity** |
| VM | -0.130 | -0.028 | **Infinity** | **0.409** | **0.399** | **Infinity** |
| WH | **0.042** | **0.077** | **Infinity** | **0.189** | **0.379** | **Infinity** |
| LP - |  |  |  |  |  |  |
| LS | **0.042** | 0.008 | **Infinity** | **0.044** | 0.043 | **Infinity** |
| MC | -0.065 | -0.086 | **90.41** | 0.044 | 0.329 | 2.87 |
| NB | -0.107 | -0.109 | **70.02** | **-** | **-** | **Infinity** |
| NW | -0.032 | -0.042 | **139.89** | **0.243** | 0.123 | **9.00** |
| SB | -0.005 | -0.011 | 65.42 | **0.074** | -0.014 | **Infinity** |
| SH | **0.082** | **0.037** | **Infinity** | **0.163** | **0.205** | **Infinity** |
| VM | -0.062 | -0.078 | **Infinity** | **0.332** | **0.503** | **19.44** |
| WH | **0.076** | **0.047** | **Infinity** | **0.063** | **0.212** | **Infinity** |
| LS - |  |  |  |  |  |  |
| MC | -0.050 | -0.045 | 43.87 | 0.040 | 0.210 | 1.73 |
| NB | -0.016 | -0.019 | **Infinity** | **-** | **-** | **-** |
| NW | -0.024 | -0.026 | **133.01** | **0.310** | 0.128 | **9.39** |
| SB | **0.026** | **0.013** | **Infinity** | **0.143** | 0.043 | **Infinity** |
| SH | **0.013** | 0.001 | **Infinity** | **0.221** | **0.216** | **Infinity** |
| VM | -0.037 | -0.025 | **68.83** | **0.393** | **0.412** | **Infinity** |
| Subpopulation | Nuclear DNA | | | Mitochondrial DNA | | |
|  | Fst | Rst | Genic differentiation (χ2) | Fst | Θst | χ2 |
| LS - WH | **0.050** | **0.042** | **Infinity** | **0.165** | **0.250** | **Infinity** |
| MC - |  |  |  |  |  |  |
| NB | **0.028** | -0.001 | **Infinity** | **-** | **-** | **Infinity** |
| NW | **0.038** | -0.004 | **Infinity** | 1.000 | 1.000 | **10.82** |
| SB | -0.048 | -0.045 | **Infinity** | **0.281** | 0.287 | **19.44** |
| SH | -0.113 | -0.082 | **Infinity** | **0.487** | **0.795** | **12.72** |
| VM | 0.011 | 0.003 | 57.46 | 1.000 | 1.000 | **10.79** |
| WH | **0.021** | 0.034 | **Infinity** | **0.328** | **0.649** | **Infinity** |
| NB - |  |  |  |  |  |  |
| NW | **0.051** | **0.036** | **Infinity** | **-** | **-** | **-** |
| SB | -0.080 | -0.049 | **72.40** | **-** | **-** | **-** |
| SH | -0.082 | -0.092 | **Infinity** | **-** | **-** | **-** |
| VM | **0.026** | 0.010 | **Infinity** | **-** | **-** | **-** |
| WH | **0.049** | 0.013 | **Infinity** | **-** | **-** | **-** |
| NW - |  |  |  |  |  |  |
| SB | -0.020 | -0.001 | **Infinity*** | **0.273** | 0.086 | **Infinity** |
| SH | -0.105 | -0.051 | **Infinity** | -0.061 | 0.165 | 0.16 |
| VM | **0.035** | **0.034** | **Infinity** | 1.000 | 1.000 | **12.53** |
| WH | **0.035** | **0.058** | **Infinity** | **0.237** | -0.046 | **10.14** |
| SB - |  |  |  |  |  |  |
| SH | **0.056** | **0.026** | **Infinity** | **0.200** | **0.164** | **Infinity** |
| Subpopulation | Nuclear DNA | | | Mitochondrial DNA | | |
|  | Fst | Rst | Genic differentiation (χ2) | Fst | Θst | χ2 |
| SB - VM | -0.047 | -0.015 | **Infinity** | **0.331** | **0.464** | **Infinity** |
| WH | **0.061** | **0.067** | **Infinity** | **0.120** | **0.224** | **Infinity** |
| SH - |  |  |  |  |  |  |
| VM | -0.108 | -0.106 | **Infinity** | **0.539** | **0.782** | **Infinity** |
| WH | -0.042 | -0.029 | **92.71** | **0.155** | **0.098** | **Infinity** |
| VM - |  |  |  |  |  |  |
| WH | **0.028** | -0.001 | **Infinity** | **0.375** | **0.596** | **Infinity** |

*Chi-square = infinity (and test is highly significant) also for test of genotypic differentiation, performed because out of HWE.
