# Supplementary material for: Implications of the Circumpolar Genetic Structure of Polar Bears for Their Conservation in a Rapidly Warming Arctic
Source: PLoS One. 2015 Jan 6;10(1):e112021. doi: 10.1371/journal.pone.0112021 (PMC4285400; doi:10.1371/journal.pone.0112021)
Supplement: S6 Table — Genetic differentiation within the Southern Hudson Bay (SH) and Davis Strait (DS) subpopulations of polar bears sampled during autumn while bears are on land. In the two-way comparison within DS: Northern indicates samples from polar bears north of Hudson Strait; Southern indicates samples south of Hudson Strait; samples from the Hudson Strait portion of the Foxe Basin subpopulation are not included in this comparison. In the three-way comparison: Southern, as above; Northern Baffin includes samples north of Frobisher Bay on Baffin Island to the border with the Baffin Bay subpopulation; Hudson Strait includes samples south of Frobisher Bay on Baffin Island and along Hudson Strait, including adjacent Hudson Strait samples from the Foxe Basin subpopulation. Akimiski Island is in James Bay, which is in the SH subpopulation; the Hudson Bay Coast is also within SH. All values are significant at α = 0.05/number of loci. (DOCX) [file pone.0112021.s012.docx]

**Table S6**. Genetic differentiation within the Southern Hudson Bay (SH) and Davis Strait (DS) subpopulations of polar bears sampled during autumn while bears are on land. In the two-way comparison within DS: Northern indicates samples from polar bears north of Hudson Strait; Southern indicates samples south of Hudson Strait; samples from the Hudson Strait portion of the Foxe Basin subpopulation are not included in this comparison. In the three-way comparison: Southern, as above; Northern Baffin includes samples north of Frobisher Bay on Baffin Island to the border with the Baffin Bay subpopulation; Hudson Strait includes samples south of Frobisher Bay on Baffin Island and along Hudson Strait, including adjacent Hudson Strait samples from the Foxe Basin subpopulation. Akimiski Island is in James Bay, which is in the SH subpopulation; the Hudson Bay Coast is also within SH. All values are significant at α = 0.05/number of loci.

| Region | F_ST_ | R_ST_ | Genic differentiation χ^2^ (df) | Genotypic differentiation χ^2^ (df) |
| --- | --- | --- | --- | --- |
| Davis Strait |  |  |  |  |
| Northern vs. Southern | 0.006 | 0.006 | Infinity (40) | Infinity (40) |
| Northern Baffin vs. Hudson Strait | 0.008 | 0.006 | Infinity (40) | Infinity (40) |
| Northern Baffin vs. Southern | 0.015 | 0.014 | Infinity (40) | Infinity (40) |
| Hudson Strait vs. Southern | 0.007 | 0.005 | Infinity (40) | Infinity (40) |
| Southern Hudson Bay |  |  |  |  |
| Akimiski Island vs. Hudson Bay Coast | 0.016 | 0.034 | 84.80 (42) | 87.33 (42) |
