# Supplementary material for: Implications of the Circumpolar Genetic Structure of Polar Bears for Their Conservation in a Rapidly Warming Arctic
Source: PLoS One. 2015 Jan 6;10(1):e112021. doi: 10.1371/journal.pone.0112021 (PMC4285400; doi:10.1371/journal.pone.0112021)
Supplement: S7 Table — Hierarchical analysis molecular variance (AMOVA) among subpopulations and various groupings of microsatellite (θCT, θSC) and mitochondrial (ΦCT,ΦSC) alleles to test hypotheses of groupings of the global subpopulations of polar bears: Baffin Bay (BB); Barents Sea (BS); Chukchi Sea (CS); Davis Strait (DS); East Greenland (EG); Foxe Basin (FB); Gulf of Boothia (GB); Kane Basin (KB); Kara Sea (KS); Laptev Sea (LP); Lancaster Sound (LS); M'Clintock Channel (MC); Northern Beaufort Sea (NB); Norwegian Bay (NW); Southern Beaufort Sea (SB); Southern Hudson Bay (SH); Viscount Melville (VM); and Western Hudson Bay (WH). Bold values are significant at α = 0.05/number of loci = 0.0023–0.003 (for microsatellite data) or 0.05 (for mtDNA data). (DOCX) [file pone.0112021.s013.docx]

**Table S7.** Hierarchical analysis molecular variance (AMOVA) among subpopulations and various groupings of microsatellite (Θ_CT,_ Θ_SC_) and mitochondrial (Φ_CT_ ,Φ_SC_) alleles to test hypotheses of groupings of the global subpopulations of polar bears: Baffin Bay (BB); Barents Sea (BS); Chukchi Sea (CS); Davis Strait (DS); East Greenland (EG); Foxe Basin (FB); Gulf of Boothia (GB); Kane Basin (KB); Kara Sea (KS); Laptev Sea (LP); Lancaster Sound (LS); M’Clintock Channel (MC); Northern Beaufort Sea (NB); Norwegian Bay (NW); Southern Beaufort Sea (SB); Southern Hudson Bay (SH); Viscount Melville (VM); and Western Hudson Bay (WH). Bold values are significant at α = 0.05/number of loci = 0.0023 – 0.003 (for microsatellite data) or 0.05 (for mtDNA data).

|  |  | Microsatellite data | | Mitochondrial data§ | |
| --- | --- | --- | --- | --- | --- |
| Hypothesis | Description | Θ_CT_ (% of variance) | Θ_SC_ (% of variance) | Φ_CT_ (% of variance) | Φ_SC_ (% of variance) |
| A. Based on structure, *Pritchard’s Method** of assessing clusters | 3 Clusters:   - Polar Basin (BS, KS, LP, SB, CS, NB) - Canadian Archipelago (VM, MC, GB, LS, NW, KB, BB and northern DS) - Southern Canada (WH, SH, FB and southern DS) | **0.033 (3.27), P < (0.0001)** | -0.050 (-4.83), P = 1.000 | **0.089 (8.93), P = 0.012** | **0.131 (11.93), P < 0.0001** |
| B. Based on structure, *Pritchard’s Method** of assessing clusters, with division of the Polar Basin | 4 Clusters:   - Eastern Polar Basin (BS, KS, LP) - Western Polar Basin (SB, CS, NB) - Canadian Archipelago (VM, MC, GB, LS, NW, KB, BB and northern DS) - Southern Canada (WH, SH, FB and southern DS) | 0.006 (0.58), P = 0.292 | -0.030, P = 1.000 | **0.087 (8.73), P = 0.0087** | **0.128 (11.72), P < 0.001** |
| C. Based on structure, *Evanno’s Method*† of assessing clusters | 2 Clusters:   - Polar Basin (BS, KS, LP, SB, CS, NB) - Eastern Arctic and Subarctic of Canada (VM, MC, GB, LS, NW, KB, BB, DS, WH, SH, FB) | 0.024 (2.44), P = 0.006 | 0.038 (-3.75), P = 1.00 | **0.092 (9.16), P = 0.0244** | **0.157 (14.30), P < 0.0001** |
| D. Based on proposed Canadian Conservation Units^‡^ | 4 Clusters:   - Central Arctic (BB, KB, LS, MC, GB, VM) - Polar Basin (NB, SB, CS, LP, KS, BS, EG) - Davis Strait - Hudson Bay (SH, WH, FB) | 0.023 (2.26), P = 0.022 | -0.042 (-4.10), P = 1.00 | **0.074 (7.40), P = 0.029** | **0.137 (12.71), P < 0.0001** |

*(1)

†(2)

‡Groupings based on Thiemann et al.’s (3) assessment of subpopulations, which occur in whole or in part within Canada, but expanded to include all circumpolar subpopulations. NW was designated the sole subpopulation in the Thiemann et al.’s (3) “High Arctic” grouping, but was not included in this AMOVA, because there would be no variation within the group.

§Groups formed from mtDNA data do not include the subpopulations: NW, NB, EG, KB

LITERATURE CITED

1. Pritchard JK, Stephens M, Donnelly P (2000) Inference of population structure using multilocus genotype data. Genetics 155:645-959.

2. Evanno G, Regnaut S, Goudet J (2005) Dectecting the number of clusters of individuals using the software structure: a simulation study. Molec Ecol 14:2611-2620.

3. Thiemann GW, Derocher AE, Stirling I (2008) Polar bear *Ursus maritimus* conservation in Canada: an ecological basis for identifying designatable units. Oryx 42(4):504-515.
