# Supplementary material for: Implications of the Circumpolar Genetic Structure of Polar Bears for Their Conservation in a Rapidly Warming Arctic
Source: PLoS One. 2015 Jan 6;10(1):e112021. doi: 10.1371/journal.pone.0112021 (PMC4285400; doi:10.1371/journal.pone.0112021)
Supplement: S8 Table — The proportion of non-migrants (95% CI) over the last ca. 1–3 generations for four genetic clusters of polar bears calculated using program bayesass (1). The Eastern Polar Basin Cluster includes polar bears from East Greenland, Barents Sea, Kara Sea and Laptev Sea subpopulations. The Western Polar Basin Cluster includes polar bears from the Chukchi Sea, Southern Beaufort Sea and Northern Beaufort Sea subpopulations. The Canadian Archipelago Cluster includes Viscount Melville, M'Clintock Channel, Gulf of Boothia, Lancaster Sound, Norwegian Bay, Kane Basin, Baffin Bay MUs, and the region north of Hudson Strait in the Davis Strait subpopulations. The Southern Canada Cluster includes Foxe Basin, Southern Hudson Bay, Western Hudson Bay MUs, and the region south of Hudson Bay in the Davis Strait subpopulations. Theta (θ) for each cluster is calculated from microsatellite (effective population size (Ne), scaled to mutation rate (µ) and mtDNA data (female effective population size (Nf), scaled to mutation rate) using the program MIGRATE. (DOCX) [file pone.0112021.s014.docx]

**Table S8.** The proportion of non-migrants (95% CI) over the last ca. 1 – 3 generations for four genetic clusters of polar bears calculated using program bayesass (1). The Eastern Polar Basin Cluster includes polar bears from East Greenland, Barents Sea, Kara Sea and Laptev Sea subpopulations. The Western Polar Basin Cluster includes polar bears from the Chukchi Sea, Southern Beaufort Sea and Northern Beaufort Sea subpopulations. The Canadian Archipelago Cluster includes Viscount Melville, M’Clintock Channel, Gulf of Boothia, Lancaster Sound, Norwegian Bay, Kane Basin, Baffin Bay MUs, and the region north of Hudson Strait in the Davis Strait subpopulations. The Southern Canada Cluster includes Foxe Basin, Southern Hudson Bay, Western Hudson Bay MUs, and the region south of Hudson Bay in the Davis Strait subpopulations. Theta (θ) for each cluster is calculated from microsatellite (effective population size (N_e_), scaled to mutation rate (µ) and mtDNA data (female effective population size (N_f_), scaled to mutation rate) using the program migrate.

| Cluster | Proportion non-migrants | θ (N_e_µ) | θ (N_f_µ) |
| --- | --- | --- | --- |
| Eastern Polar Basin | 0.941 (0.888–0.993) | 0.987 (0.929–1.046) | 0.061 (0.046–0.082) |
| Western Polar Basin | 0.678 (0.657–0.699) | 1.008 (0.954–1.064) | 0.007 (0.006–0.009) |
| Canadian Archipelago | 0.699 (0.621–0.777) | 0.983 (0.944–1.022) | 0.007 (0.006–0.008) |
| Southern Canada | 0.952 (0.912–0.991) | 0.941 (0.905–0.980) | 0.005 (0.005–0.006) |

LITERATURE CITED

1. Wilson GA & Rannala B (2003) Bayesian inference of recent migration rates using multilocus genotypes. Genetics 163:1177-1191.
