# Supplementary material for: Implications of the Circumpolar Genetic Structure of Polar Bears for Their Conservation in a Rapidly Warming Arctic
Source: PLoS One. 2015 Jan 6;10(1):e112021. doi: 10.1371/journal.pone.0112021 (PMC4285400; doi:10.1371/journal.pone.0112021)
Supplement: S9 Table — Coalescent times (thousands of years ago) to most recent common ancestor for major mitochondrial lineages of brown and polar bears based on 581 bp of the mitochondrial DNA control region, excluding indels. Provided are nodal support for lineages based on Bayesian analysis in BEAST and median age of nodes with 95% confidence intervals (CI). ABC bears refer to brown bears from the Alexander Archipelago, Alaska, USA. (DOCX) [file pone.0112021.s015.docx]

**Table S9**. Coalescent times (thousands of years ago) to most recent common ancestor for major mitochondrial lineages of brown and polar bears based on 581bp of the mitochondrial DNA control region, excluding indels. Provided are nodal support for lineages based on Bayesian analysis in BEAST and median age of nodes with 95% confidence intervals (CI). ABC bears refer to brown bears from the Alexander Archipelago, Alaska, USA.

| Lineage | Node support | Node age (kya) | Age 95% CI (kya) |
| --- | --- | --- | --- |
| All polar bears | 0.895 | 64 | 20-149 |
| All brown and polar bears | N/A | 394 | 143-925 |
| Western and Eastern Beringian brown bears | 0.997 | 161 | 40-399 |
| ABC brown bears | 0.998 | 27 | 4-77 |
| ABC brown bears and all polar bears | 0.810 | 92 | 31-164 |
| ABC, polar, and Poolepynten fossil bears* | 0.996 | 143 | 120-248 |

*(1)

LITERATURE CITED

1. Miller W, Schuster SC, Welch AJ, Ratan A, Bedoya-Reina OC, et al. (2012) Polar and brown bear genomes reveal ancient admixture and demographic footprints of past climate change. Proc Natl Acad Sci U S A - Plus. 109(36):E2382-2390.
