# Supplementary material for: Implications of the Circumpolar Genetic Structure of Polar Bears for Their Conservation in a Rapidly Warming Arctic
Source: PLoS One. 2015 Jan 6;10(1):e112021. doi: 10.1371/journal.pone.0112021 (PMC4285400; doi:10.1371/journal.pone.0112021)
Supplement: S10 Table — Average pairwise distances within and among haplotypes from polar bears sampled from 15 subpopulations, the ancient Poolypenten (GenBank Accession No. GU573488)* polar bear and haplotypes found within the three clades of Alaskan brown bears. Values were generated using the Tamura-Nei (I+G0.69) model of substitution. (DOCX) [file pone.0112021.s016.docx]

**Table S10.** Average pairwise distances within and among haplotypes from polar bears sampled from 15 subpopulations, the ancient Poolypenten (GenBank Accession No. GU573488)* polar bear and haplotypes found within the three clades of Alaskan brown bears. Values were generated using the Tamura-Nei (I+G_0.69_) model of substitution.

|  | Modern polar bear | Poolypenten polar bear | Western Beringian brown bear clade | Eastern Beringian brown bear clade |
| --- | --- | --- | --- | --- |
| Modern polar bear |  |  |  |  |
| Poolypenten polar bear | 0.012 |  |  |  |
| ABC Brown bear | 0.015 | 0.012 |  |  |
| Western Beringian brown bear clade | 0.060 | 0.060 | 0.063 |  |
| Eastern Beringian brown bear clade | 0.058 | 0.056 | 0.062 | 0.031 |

*(1)

LITERATURE CITED

1. Miller W, Schuster SC, Welch AJ, Ratan A, Bedoya-Reina OC, et al. (2012) Polar and brown bear genomes reveal ancient admixture and demographic footprints of past climate change. Proc Natl Acad Sci U S A - Plus. 109(36):E2382-2390.
