# Supplementary material for: Implications of the Circumpolar Genetic Structure of Polar Bears for Their Conservation in a Rapidly Warming Arctic
Source: PLoS One. 2015 Jan 6;10(1):e112021. doi: 10.1371/journal.pone.0112021 (PMC4285400; doi:10.1371/journal.pone.0112021)
Supplement: S12 Table — Permissions and permits for collection of tissue samples of polar bears used in this study. (DOCX) [file pone.0112021.s018.docx]

**Table S12.** Permissions and permits for collection of tissue samples of polar bears used in this study.

| Subpopulation | Genetic data previously published? | Years | Type of collection | Jurisdiction | Permissions for sample collection |
| --- | --- | --- | --- | --- | --- |
| FB | (1) | 1991, 1994 - 97 | Harvest | Nunavut -NWT | Permitted by Government of Nunavut^1^ |
| MC | (1) | 1996 | Harvest | Nunavut - NWT | Permitted by Government of Nunavut^1^ |
| BB | (1) | 1995 | Capture | Nunavut - NWT | Permitted by Government of Nunavut^1^ |
| BS | (1, 2) | 1986, 1990 - 2006 | Capture | Norway | Norwegian Animal Care Unit Permits: 1997/00755-006/MA/311.2(1997 - 2003); S-2004-9887-1 (2004 - 2005); 2006-97 (2006); Letters from Government of Svalbard (1990 - 1992; 1994 - 1996) and Norwegian Animal Research Authority (1995 - 1999) |
| CS* | (1) | 1986-1994 | Capture | Russia | No permits found |
| DS | (1) | 1991, 1993 - 94 | Capture | Canada | Permitted by Canada^3^ |
| EG | (1) | 1990 | Capture | Greenland | Permitted by Greenland^2^ |
| GB | (1) | 1995 - 96 | Capture | Nunavut - NWT | Permitted by Government of Nunavut^1^ |
| KB | (1) | 1992 - 96 | Capture | Nunavut - NWT | Permitted by Government of Nunavut^1^ |
| KS* | (1) | 1991, 1994 - 95 | Capture | Russia | No permits found |
| LP* | (1) | 1993 - 94 | Capture | Russia | No permits found |
| LS | (1) | 1985, 1993 - 96 | Capture | Nunavut - NWT | Permitted by Government of Nunavut^1^ |
| MC | (1) | 1994 - 96, 2000 | Capture | Nunavut - NWT | Permitted by Government of Nunavut^1^ |
| Subpopulation | Genetic data previously published? | Years | Type of collection | Jurisdiction | Permissions for sample collection |
| NB | (1) | 1986 - 87; 1993 | Capture | Canada | Permitted by Canada^3^ |
| NW | (1) | 1993, 1995 - 96 | Capture | Nunavut - NWT | Permitted by Government of Nunavut^1^ |
| SB* | (1) | 1982 - 1994; 1996 - 1999; 2001 - 04; 2009 - 2010 | Capture | U.S. | U.S. Marine Mammal Permits 690038 (for 1999 - 2010); no permits found before this time; U.S. Geological Survey Animal Care and Use Committee at Alaska Science Center started in 2006: Numbers 061214-2 (2006 - 2009) and Number 2010-3 (2010) |
| VM | (1) | 1973, 1989, 1991 - 92 | Capture | Nunavut - NWT | Permitted by Government of Nunavut^1^ |
| WH | (1) | 1986 - 90, 1994 - 95 | Capture | Canada | Permitted by Canada^3^ |
| BB | New | 1999, 2003, 2006 - 08, 2011 | Harvest | Nunavut | Nunavut WRP 2006-000878; 2007-000893; 2008-1014; 2003-183 |
| DS | New | 2008 | Harvest | Nunavut | Nunavut WRP 2008-1014 |
| FB | New | 2006, 2008 - 10 | Harvest | Nunavut | Nunavut WRP 2006-000878; 2008-1014; Permitted by Government of Nunavut^1^ |
| GB | New | 2008 | Harvest | Nunavut | Nunavut WRP 2008-1014 |
| LS | New | 2003, 2008, 2010 | Harvest | Nunavut | Nunavut WRP 2003-183; 2008-1014 and Permitted by Government of Nunavut^1^ |
| MC | New | 2008 | Harvest | Nunavut | Nunavut WRP 2008-1014 |
| NW | New | 2008 | Harvest | Nunavut | Nunavut WRP 2008-1014 |
| VM | New | 2008 | Harvest | Nunavut | Nunavut WRP 2008-1014 |
| WH | New | 2003, 2008 | Harvest | Nunavut | Nunavut WRP 2003-183; 2008-1014 |
| Subpopulation | Genetic data previously published? | Years | Type of collection | Jurisdiction | Permissions for sample collection |
| DS | New | 2005-07 | Capture | Nunavut | Nunavut WRP 2005-000757; 2006-000868; 2007-000890 |
| BB | New | 2005 - 07 | Capture | Nunavut | Nunavut WRP 2005-000757; 2006-000868; 2007-000890 |
| CS | New | 2008 -11 | Capture | U.S. | U.S. Fish and Wildlife Service Region 7 Institutional Animal Care and Use Committee Number 2013001 |
| FB | New | 2009 | Capture | Nunavut | Permitted by Government of Nunavut^1^ |
| SH | New | 2007 - 2009 | Capture | Ontario | Ontario Ministry of Natural Resources Permit 95-07, -08, -09 |
| FB | New | 2008 | Remote Biopsy | Nunavut | Nunavut WRP 2008-1024 |
| CS | New | 2008 - 11 | Shed hair | Russia | No permits (A. Boltunov, personal communication) |
| LP | New | 2008 - 11 | Shed hair | Russia | No permits (A. Boltunov, personal communication) |
| *note some older samples were not used in Paektau et al. 1999 (1), but extracted/analyzed as 'new' for this present study | | | | | |
| ^1^The data and samples (collected 1985 - 2010) included in this manuscript were collected in the (now) territory of Nunavut, Canada. They were obtained from polar bear capture studies and studies of legally harvested bears; these studies were permitted by the Wildlife Research Section of the (now) Government of Nunavut (D. Gissing, Director of Wildlife Management, Government of Nunavut) | | | | | |
| ^2^ The samples from East Greenland were collected according to the Greenland regulations for protection of polar bears (Anon. 1992) and animal care.^.^Anonymous 1992. Hjemmestyrets bekendtgørelse nr. 25 af 2. oktober 1992 om fredning af isbjørne i Grønland. Offentliggørelse af Hjemmestyrets bekendtgørelser. Grønlands Hjemmestyre: 5 pp. (Executive order of the Greenland Home Rule No. 25 of 2 October 1992 concerning protection of polar bears in Greenland: 5 p.) (In Danish) | | | | | |
| ^3^All handling procedures were reviewed annually and approved by the Prairie and Northern Region, Environment Canada, Animal Care Committee; no permit number (I. Stirling, personal communication) | | | | | |

LITERATURE CITED

1. Paetkau D, Amstrup SC, Born EW, Calvert W, Derocher AE, et al. (1999) Genetic structure of the world's polar bear populations. Molec Ecol 8: 1571-1584.

2. Zeyl E, Aars J, Ehrich D, Wiig Ø (2009) Families in space: relatedness in the Barents Sea population of polar bears (*Ursus maritimus*). Molec Ecol 18: 735-749.
