# Supplementary material for: Correction: Implications of the circumpolar genetic structure of polar bears for their conservation in a rapidly warming Arctic
Source: PLoS One. 2015 Aug 14;10(8):e0136126. doi: 10.1371/journal.pone.0136126 (PMC4537302; doi:10.1371/journal.pone.0136126)
Supplement: S1 Supporting Information — (DOCX) [file pone.0136126.s001.docx]

**Supplementary Information: Materials and Methods**

*Sampling.* We combined microsatellite data from samples collected between 1973 and 2006 (n = 869) and published in previous studies [1-3], with new data generated from samples (n = 1,879) collected between 1982 and 2012 (Figure S1a, Table S11). In earlier global assessments of polar bear microsatellite genetic structure, Paetkau et al. [2] and Cronin and MacNeil [4] analyzed 473 samples from portions of 17 of the 19 subpopulations. Here, we additionally sample the Southern Hudson Bay subpopulation (SH; Figure S1a, Table S11). Further, we increase the quantity and geographic breadth of sampling from 10 subpopulations. Notably, we include samples from a large portion of Davis Strait (DS; i.e., north of Hudson Strait), which was not sampled in the first circumpolar analysis [2, 4], although this is the portion of DS sampled by Crompton et al. [5] in a regional genetic survey. The previous circumpolar effort [2, 4] used samples collected up until 1995; we analyze more recent samples for 12 of the global subpopulations. In total, we analyze 157 from the 1980s, 623 from the 1990s, 1,782 from the 2000s and 186 from the 2010s (Figure S1a; Table S11). We also tracked the origins of published and new samples and corrected latitude and longitude data, which changed subpopulation designations for a few samples (with respect to the previous effort) and further eliminated samples that were from dependent animals (i.e., if a mother and her cub were harvested or captured together). Four hundred and eleven samples from 15 subpopulations were available for mtDNA analysis (Figure S1b, Table S11).

*Laboratory Techniques.* DNA extraction, PCR amplification and genotyping protocols for previously published data are described in the literature [1, 2]. For new samples, DNA was extracted, amplified and genotyped at the US Geological Survey Alaska Science Center (n = 538; USGS) or Wildlife Genetics International (n *=* 1,341; WGI), or was extracted at WGI and amplified and genotyped at USGS (n = 107).

At WGI, new microsatellite genotypes for the DS, Baffin Bay (BB), and Foxe Basin (FB) subpopulations were determined at 20 microsatellite loci following protocols described in Paetkau et al. [6] and Kendall et al. [7]. At the USGS, additional genotypes were determined at 22 microsatellite DNA loci with PCR primers developed previously [3, 8-11] for samples from the following subpopulations: Chukchi Sea (CS), Gulf of Boothia (GB), Laptev Sea (LP), Lancaster Sound (LS), Southern Beaufort Sea (SB), SH and Western Hudson Bay (WH). PCR amplifications and agarose electrophoresis of PCR products followed DNA extraction. We used microchecker [12] to identify potential genotyping errors. We combined published mtDNA data from the Barents Sea subpopulation (BS; n *=* 30; [13]) with new mtDNA sequences collected at USGS from 14 additional subpopulations (n *=* 381), including amplification of a portion of the mtDNA cytochrome *b* gene, the tRNA^thr^ and tRNA^pro^ genes, and the hypervariable region of the control region [14]. PCR amplifications, cycle-sequencing protocols, and post-sequencing processes followed Jackson et al. [15]. To check for genotyping errors, 10–15% of the samples were extracted, amplified, and genotyped or sequenced in duplicate. We used sterile technique in the handling of all DNA, and all PCR procedures were done with positive and negative controls to verify amplification without contamination. To standardize alleles at microsatellite loci across datasets developed in different labs, genotypes were calibrated against DNA standards of known size, provided by DP and EZ. MtDNA sequence data from brown bears generated in a previous study [16], were used as an outgroup to root relationships among polar bear haplotypes within a phylogenetic evolutionary context.

*Analysis.*

*Genetic Diversity.* We quantified genetic variation (mean number of alleles per locus (A) observed heterozygosity (H_o_) and expected heterozygosity (H_e_)) using biosys [17] and the microsatellite toolkit (Version 2.1, [18]) computer programs. Because sample sizes were disparate, we also calculated allelic richness using f-stat version 2.9.3.2 [19]. All microsatellite loci were tested for gametic phase disequilibrium and for deviations from Hardy-Weinberg equilibrium (HWE) using the Fisher’s Exact Test in genepop version 3.3 [20]. F_IS_ was calculated using f-stat. For all multiple tests, we corrected significance values using a serial Bonferroni approach [21].

MtDNA control region haplotypes were assigned based on at least a single nucleotide substitution or insertion/deletion (indel) found within the segment sequenced. We used arlequin 2.0 [22] to estimate haplotype (*h*) and nucleotide (π) diversity ([23]; Eq. 8.4 and 10.6, respectively). Indels were coded as transitions to avoid underestimation in diversity metrics. We used the Ewens-Watterson (Eν; [24, 25]) to test for selective neutrality of the mtDNA sequences, obtained using arlequin.

*Decadal Comparisons.* We calculated the χ^2^ distribution of alleles between each decadal group [20] and further compared the allele frequency distributions using F-statistics [26]. We used Bayesian clustering (baps version 3.2; [27]) to infer the occurrence of population structure, based on microsatellite frequency data, among individuals sampled during different decades without *a priori* knowledge of sampling decade.

*Genetic Differentiation.* Overall estimates of F_ST_ variance for the microsatellite data were obtained using f-stat, and a maximum possible value of F_ST_ was calculated using recodedata [28]. Estimates of inter-subpopulation F_ST_ variance were derived using arlequin [22]; significance of F_ST_ was based on random permutation tests (n = 1,000), in which alleles were randomly permuted between subpopulations. We also applied the Fisher’s Exact test [26] and α = 0.05, for comparison to Paetkau et al. [2].

We used the maximum likelihood criterion in modeltest 3.06 [29] to determine the evolutionary model that best fit the mtDNA sequence data. The resulting genetic distances were used to calculate Φ [29], which tests for inter-subpopulation variance, at the mtDNA locus, and tested for significance using arlequin [21]. We tested for significance of heterogeneity of microsatellite alleles and mtDNA haplotypes between subpopulations with an exact test as described in Raymond and Rousset [20], using genepop. To address previous hypotheses about within-subpopulation substructure in DS [31] and SH [5], we used the above tests to examine genetic differentiation between locales within each subpopulation.

We used hierarchical analyses of molecular variance (AMOVA; [22]) to test for significance of geographic partitioning of hypothesized genetic units with microsatellite and mtDNA loci. To test proposed conservation units [32], we categorized 4 regions: the Central Arctic, the Polar Basin, Davis Strait, and Hudson Bay (Table S5, Hypothesis D). We also explored various *a posteriori* groupings (Hypotheses A–C; Table S5), testing clusters identified in structure analyses of microsatellite loci. We assumed that groupings maximizing values of Φ*_CT_* and that were significantly different from random distributions of individuals were the most probable geographical subdivisions. Thus, given concordance between the distribution of genetic subdivisions at the neutral genetic markers and subspecies delineations, Φ*_CT_* should be significant, and account for more among-group variation than for alternative groupings.

*Estimation of Gene Flow*. Estimates of gene flow among the four clusters of polar bears, identified by structure and biological rationale, were calculated in bayesass, version 3.0.1 [33] and migrate, version 3.0.3 [34, 35]. These programs use different models to estimate gene-flow rates. bayesass uses an assignment method and does not incorporate genealogy, whereas migrate uses a coalescent model of population differentiation that incorporates two parameters scaled to the mutation rate (μ): θ, the effective population size parameter (4N_e_μ); and M, the rate of gene flow (m/μ), where m is the number of effective migrants per generation. These estimates of gene flow can be interpreted differently and at different temporal scales. bayesass reflects gene flow that occurred only in the past 1-3 generations, whereas in migrate gene-flow estimates are averaged over the past *n* generations, where *n* equals the number of generations in which the clusters have been at mutation–drift equilibrium. Because we wanted input dataset for the gene flow analyses of microsatellite data to 1) be comparable for both MIGRATE and BayesAss analyses; 2) avoid as much as possible the use of individuals with large amounts of missing data; and because 3) 15 individuals are recommended (for efficiency) for the MIGRATE analyses (P. Beerli and M. Kuhner, pers. comm. to SAS), and we wanted each area to be fairly equally represented, we randomly sampled individuals from strata (geographic areas) within each subpopulation, selecting from 26 (WP), 34 (EP) and 60 (CA and SC), depending on the number available for analysis.

bayesass analysis was conducted with the microsatellite data set using the default delta values for allelic frequency, migration rate, and inbreeding. Subsequent analyses incorporated different delta values to ensure that proposed changes between chains at the end of the run were between 20% and 40% of the total chain length [33]. Once the delta values (ΔA = 0.70, Δm = 0.40, and ΔF = 0.85) were within the accepted proportion of proposed changes (A = 38%, m = 27%, and F = 37%), analyses were conducted three additional times (50 million iterations, ten million burn-in, and sampling frequency of 2,000) with different random seeds. All parameter estimates converged.

The number of migrants per generation (*N_e_m*) for nuclear microsatellite and number of female migrants per generation (*N_f_m*) for mtDNA were calculated using migrate. We ran migrate using maximum likelihood search parameters; ten short chains (1,000 used trees out of 20,000 sampled), five long chains (10,000 used trees out 200,000 sampled), and five adaptively heated chains (start temperatures: 1, 1.5, 3, 6, and 12; swapping interval = 1). We ran models three times to ensure the convergence of parameter estimates. The alternative model was evaluated for goodness-of-fit given the data using a log-likelihood ratio test [35].

*Phylogenetic Analyses of MtDNA Sequences.* Phylogenetic comparisons were made using a dataset comprised of haplotypes observed among 15 subpopulations of polar bears. We also included 37 haplotypes from 144 individuals representing the three Alaskan brown bear clades [15] to root the tree. Phylogenetic analyses of control region sequences were conducted using paup*4.0b8 [36], using maximum parsimony (MP), maximum likelihood (ML) and distance (minimum evolution, ME) approaches. The nucleotide substitution model that best fit the data was the Tamura-Nei model [37], incorporating a gamma distribution (γ = 0.6886) and the proportion of invariable sites (I = 0.8748), as determined in modeltest under the Akaike Information Criterion (AIC). The transition to transversion ratio was estimated at 58.77. We thus weighted tranversions 59:1 over transitions in subsequent analyses where appropriate. We used the TrN+I+G model in maximum likelihood ML and ME tree reconstructions. Heuristic tree searches were conducted for each analysis, with 20 and 100 random additions of taxa for ML and MP analyses, respectively, each followed by tree bisection-reconnection topological rearrangements. We assessed robustness of nodes using tree reconstructions of bootstrap-resampled data sets for 1,000 replicates under ME and MP criteria, and 200 replicates for ML criteria. We constructed an unrooted reduced median haplotype network [38] for mtDNA control region in network 4.510 (Fluxus Technology Ltd. 2009), to illustrate possible reticulations due to homoplasy.

*Changes in Historical Population Size.* Using the mtDNA data set, we assessed historical signatures of population growth within subpopulations and within larger regional groupings using the distribution of pairwise sequence differences (mismatch distributions; [39]) and the raggedness index (*rg*) of the observed distribution [40]. Timing and extent of population size change was investigated using extended Bayesian skyline plots (EBSPs; [41]). Additional evidence of historical population expansion at the subpopulation and larger cluster level was inferred from the shape of phylogenetic trees, neutrality tests sensitive to population fluctuations (Tajima's D and Fu's F_S_; [42, 43]) and comparison of diversity indices (haplotype diversity [*h*] and nucleotide diversity [π]). All statistics were calculated using DnaSP [44], testing significance of expansion measures with 10,000 coalescent simulations. High *h* coupled with low π indicates an excess of unique haplotypes reflecting unfixed point mutations, often as a result of a recently expanded population [45]. Significantly negative departures from zero for Tajima’s *D* and Fu’s F*_S_* values may indicate population expansions [42, 43, 46]. Critical significance values of 5% require a *P*-value below 0.02 [42]. We used a coalescent-based simulation method, implemented in fluctuate to further test for evidence of population expansion: ten short chains (sampling increments of ten with 1,000 steps per chain); ten long chains (sampling increments of ten with 20,000 steps per chain); a random starting tree; and a starting value of *g* set to 1. Because standard deviations are only approximate, and computations may show an upward bias, we used *g* to indicate population growth if *g* > 3 SD(*g*).

To estimate the time since population expansion, we used mismatch distributions and the nonlinear least-squares approach, applying the algorithm τ = 2*u*t, where t = the number of generations elapsed between initial population and the current population; *u* = 2μk; μ = the mutation rate per million years; and k = the length of the sequence [45]. This algorithm assumes population expansion. For estimation of mutation rate we used a coalescent Bayesian framework and included control region haplotype sequences of representative brown and polar bears as used for phylogenetic tree estimation. Parameters were set in beauti, part of the beast v1.6.1 software package [46]. Substitution model was estimated in MrModeltest v2.3 [47] under AIC to be HKY+I+G, and set to use empirical base frequencies and partitions by codon positions. With a coalescent tree prior, assuming constant population size through time, we used a relaxed clock in accordance with a lognormal distribution with uncorrelated rates. We then set the mutation rate to be estimated considering all sequences with a tip date of zero (sampled at present considering an evolutionary timescale) except for the ancient Poolepynten fossil sequence for which we applied a tip date of 120 kya [48]. We ran two independent analyses in beast for the whole control region dataset, and again having removed the region containing indels. Results were minimally different from the two runs and we report data based on sequences with indels removed to be consistent with previous studies. Analyses ran for 50 M generations, logging trees every 5,000. Runs were assessed for stationarity and convergence with trace files and ESS values (>300) in tracer v1.4 [49]. Tree files were annotated with TreeAnnotator (part of the beast software package) and nodal support for major lineages as well as node ages with associated 95% confidence intervals were retrieved from the resulting consensus tree file.

EBSPs were similarly calculated in beast [46]. All partitions were unlinked, and we used empirical base frequencies under the assumption of a relaxed clock (uncorrelated log-nor­mal). A control region mutation rate of 11% per million years, as calculated above, was applied. All priors and operators were maintained at their default values except for the changes specific to this method (EBSP tutorial and script; <http://beast.bio.ed.ac.uk/Tutorials>, downloaded 21 February 2012). Analyses were restricted to regional polar bear clusters. Each analysis was run twice for an MCMC chain length of 100 million, sampling every 10,000 states. Runs were combined in LogCombiner, part of the beast software package, and assessed for stationarity and convergence with trace files and ESS values (> 300) in tracer. EBSPs were produced from output data in Microsoft Excel 14.1.3.

LITERATURE CITED

1. Zeyl E, Aars J, Ehrich D, Wiig Ø (2009) Families in space: relatedness in the Barents Sea population of polar bears (*Ursus maritimus*). Mol Ecol 18: 735-749.
2. Paetkau D, Amstrup SC, Born EW, Calvert W, Derocher AE, et al. (1999) Genetic structure of the world's polar bear populations. Mol Ecol 8: 1571-1584.
3. Paetkau D, Calvert W, Stirling I, Strobeck C (1995) Microsatellite analysis of population structure in Canadian polar bears. Molec Ecol 4: 347-354.
4. Cronin MA, MacNeil MD (2012) Genetic relationships of extant brown bears (*Ursus arctos*) and polar bears (*Ursus maritimus*). J Hered 103: 873-881.
5. Crompton AE, Obbard ME, Petersen SD, Wilson PJ (2008) Population genetic structure in polar bears (*Ursus maritimus*) from Hudson Bay, Canada: Implications of future climate change. Biol Conserv 141: 2528-2539.
6. Paetkau D (2003) An empirical exploration of data quality in DNA-based population inventories. Mol Ecol 12: 1375-1387.
7. Kendall KC, Stetz JB, Boulanger J, MacLeod A, Paetkau D (2009) Demography and genetic structure of a recovering grizzly bear population. J Wildl Manage 73: 3-17.
8. Ostrander EA, Sprague GF, Rine J (1993) Identification and characterization of dinucleotide repeat (CA)n markers for genetic-mapping in dog. Genomics 16: 207-213.
9. Paetkau D, Strobeck C (1994) Microsatellite analysis of genetic-variation in black bear populations. Molec Ecol 3: 489-495.
10. Paetkau D, Waits LP, Clarkson PL, Craighead L, Strobeck C (1997) An empirical evaluation of genetic distance statistics using microsatellite data from bear (Ursidae) populations. Genetics 147: 1943-1957.
11. Taberlet P*,* Camarra JJ, Griffin S, Uhrès E, Hannote O, et al. (1997) Noninvasive genetic tracking of the endangered Pyrenean brown bear population. Mol Ecol 6: 869-876.
12. van Oosterhout C, Weetman D, Hutchinson WF (2006) Estimation and adjustment of microsatellite null alleles in nonequilibrium populations. Mol Ecol Notes 6: 255-256.
13. Zeyl, E, Erich D, Aars J, Bachmann L, Wiig Ø (2010) Denning-area fidelity and mitochondrial DNA diversity of female polar bears (*Ursus maritimus*) in the Barents Sea. Can J Zool 88: 1139-1148.
14. Wakeley J (1993) Substitution rate variation among sites in hypervariable region-1 of the human mitochondrial DNA. J Mol Evol 37: 613-623.
15. Jackson JV, Talbot SL, Farley S (2008) Genetic characterization of Kenai brown bears (*Ursus arctos*): microsatellite and mitochondrial DNA control region variation in brown bears of the Kenai Peninsula, southcentral Alaska. Can J Zool 86: 756-764.
16. Talbot SL, Sonsthagen SA, Sage GK, Farley SD, Dawson NG, et al. 2012. Are island brown bears isolated? Insularity and gene flow among coastal populations in southeast Alaska. J Mamm: In Press.
17. Swofford DL, Selander RB (1981) Biosys-1 - A FORTRAN program for the comprehensive analysis of electrophoretic data in population genetics and systematics. J Hered 72: 281-283.
18. Parks SDE (2001) Trypanotolerance in West African Cattle and the population genetic effects of selection. Ph.D. (University of Dublin).
19. Goudet J (1995) FSTAT (Version 1.2): A computer program to calculate F-statistics. J Hered 86: 485-486.
20. Raymond M, Rousset F (1995) An exact test for population differentiation. Evolution 49:1280-1283.
21. Rice WR (1989) Analzying tables of statistical tests. Evolution 43(1): 223-225.
22. Excoffier L, Laval G, & Schneider S (2005) Arlequin ver. 3.0: An integrated software package for population genetics data analysis. Evol Bioinform Online 1:47-50.
23. Nei M (1987) Molecular evolutionary genetics. New York: Columbia University Press.
24. Ewens WJ (1972) The sampling theory of selectively neutral alleles. Theor Popul Biol 3: 87-112.
25. Watterson G (1975) On the number of segregating sites in genetical models without recombination. Popul Biol 7: 256-276.
26. Weir BS, Cockerham CC (1984) Estimating F-statistics for the analysis of population structure. Evolution 38: 1358-1370.
27. Corander J, Marttinen P (2006) Bayesian identification of admixture events using multi-locus molecular markers. Molec Ecol 15: 2833-2843.
28. Meirmans PG (2006) Using the AMOVA framework to estimate a standardised genetic differentiation measure. Evolution 60: 2399-2402.
29. Posada D, Crandall KA (1998) modeltest: testing the model of DNA substitution. Bioinformatics 14: 817-818.
30. Excoffier L, Smouse PE, Quattro JM (1992) Analysis of molecular variance inferred from metric distances among DNA haplotypes - application to human mitochondrial DNA restriction data. Genetics 131: 479-491.
31. Peacock E, Taylor MK, Laake J, Stirling I, et al. (2013). Population ecology of polar bears in Davis Strait, Canada and Greenland. J Wildl Manage 77: 463-476.
32. Thiemann GW, Derocher AE, Stirling I (2008) Polar bear *Ursus maritimus* conservation in Canada: an ecological basis for identifying designatable units. Oryx 42: 504-515.
33. Wilson GA, Rannala B (2003) Bayesian inference of recent migration rates using multilocus genotypes. Genetics 163:1177-1191.
34. Beerli P, Felsenstein J (1999) Maximum likelihood estimation of migration rates and population numbers of two populations using a coalescent approach. Genetics 152: 763-773.
35. Beerli P, Felsenstein J (2001) Maximum likelihood estimation of a migration matrix and effective population sizes in *n* subpopulations using a coalescent approach. Proc Natl Acad Sci USA 98: 4563-4568.
36. Swofford DL (2000) PAUP*. Phylogenetic Analysis Using Parsimony (*and other methods). Sinauer Associates, Sunderland, MA, 4 p.
37. Tamura K, Nei M (1993) Estimation of the number of nucleotides substituions in the control region of mitochondrial DNA in humans and chimpanzees. Mol Biol 10:512-526.
38. Bandelt HJ, Forster P, Sykes BC, Richards MB (1995) Mitochondrial portraits of human populations using median networks. Genetics 141:743-753.
39. Rogers AR, Harpending H (1992) Population growth makes waves in the distribution of pairwise genetic differences Mol Biol Evol 9:552-569.
40. Harpending HC (1994) Signature of ancient population growth in a low resolution mitochondrial-DNA mismatch distribution. Hum Biol 66: 591-600.
41. Heled J, Drummond AJ (2008) Bayesian inference of population size history from multiple loci. BMC Evol Biol 8: 289.
42. Tajima F (1989) Statistical method for testing the neutral mutation hypothesis by DNA polymorphism. Genetics 123: 585-595.
43. Fu YX (1997) Statistical tests of neutrality of mutations against population growth, hitchhiking and background selection. Genetics 147: 915-925.
44. Librado P, Rozas J (2009) DnaSP v5: a software for comprehensive analysis of DNA polymorphism data. Bioinformatics 25: 1451-1452.

Rogers, AR (1995) Genetic evidence for a Pleistocene population explosion. Evolution 49: 608-615.

1. Drummond AJ, Rambaut, A (2007) BEAST: Bayesian evolutionary analysis by sampling trees. BMC Evol Biol 7: 214.

Nylander, JA (2004) MrModeltest v2. Program distributed by the author. Evolutionary Biology Center, Uppsala University, Uppsala, Sweden.

1. Lindqvist C, Schuster SC, Sun Y, Talbot SL, Qi J, et al. (2010) Complete mitochondrial genome of a Pleistocene jawbone unveils the origin of polar bear. Proc Natl Acad Sci U S A 107: 5053-5057.
2. Rambaut A, Drummond AJ (2007) *Tracer v1.4*, http://beast.bio.ed.ac.uk/Tracer, accessed 1 December 2009.
